# From Policy to Practice: A Global Analysis to Identify Factors That Influence the Implementation of Food-Based Dietary Guidelines by Health and Nutrition Professionals

**DOI:** 10.1016/j.cdnut.2026.109509

**Published:** 2026-08-07

**Authors:** Anna-Lena Klapp, Jonas Pardamean Simorangkir, Valentina Gallani, Daniel André Benjamin Braune, Antje Risius

**Affiliations:** 1 Department of Agricultural Economics and Rural Development, University of Göttingen, Göttingen, Germany; 2 ProVeg International, Research Department, Berlin, Germany; 3 Department of Nutrition, Fulda University of Applied Sciences, Fulda, Germany

**Keywords:** food-based dietary guidelines, FBDG, health professionals, dieticians, socioecological framework, sustainable diets, nutrition policy, policy implementation

## Abstract

**Background:**

Food-based dietary guidelines (FBDG) are essential tools for promoting healthy, sustainable diets, yet global adherence is low. Health and nutrition professionals are key to translating these policies into practice, but the factors influencing their implementation efforts remain underresearched.

**Objectives:**

This study aimed to identify factors influencing FBDG implementation from the perspective of health and nutrition professionals and to explore opportunities for strengthening implementation.

**Methods:**

Using a socioecological framework, this study employed an exploratory sequential mixed-methods design. Phase 1 involved 20 qualitative interviews with professionals across 6 continents. Insights informed phase 2, a quantitative survey (*n* = 118) of global professionals. Data were analyzed using deductive content analysis and descriptive statistics.

**Results:**

Although 80.5% of professionals use FBDG in practice, 57.6% rate them as only “somewhat” or “slightly” helpful in promoting healthy, sustainable food choices. Major barriers include perceived food industry influence (83.0%) and public misinformation. Facilitators include the “plate” visual format (77.1%) and the inclusion of more plant-based options. Critically, professionals advocate for systemic change, identifying fiscal policies—such as subsidies for healthy foods and tax reductions on vegetables and legumes—as essential to address cost barriers and create supportive food environments. Furthermore, inclusive designs that account for diverse cultural and nutritional philosophies are viewed as vital for increasing professional implementation.

**Conclusions:**

Health and nutrition professionals encounter multiple barriers and facilitators in implementing the national FBDG. Policymakers and scientific committees should address these barriers and leverage facilitators to improve FBDG development and implementation. Findings provide initial valuable insights for future research and policy efforts aimed at promoting healthy and sustainable diets.

## Introduction

In recent years, food-based dietary guidelines (FBDG) have gained increased attention across scientific disciplines as essential tools for promoting healthier and more sustainable diets [[1], [2], [3], [4], [5], [6], [7]]. Although their primary purpose is to offer dietary recommendations to the public, FBDG also underpin national food, health, and education policies. In addition, they serve as a foundational resource for health professionals—including dietitians, nutritionists, and medical doctors—who translate dietary recommendations into actionable guidance for individuals and communities [1,8]. In several countries, nutrition communication based on the national FBDG is mandatory or at least strongly encouraged for health and nutrition professionals [[8], [9], [10], [11], [12]].

Despite their central role in public health and nutrition policy, adherence to FBDG remains remarkably low worldwide. Evidence from systematic reviews, global modeling studies, and national surveys indicates that many populations fail to meet their national dietary recommendations, with common patterns including overconsumption of meat, underconsumption of vegetables, and limited public awareness or engagement with FBDG [3,[13], [14], [15], [16]].

Health professionals are frequently identified as key actors in advancing both public health and environmental goals through dietary guidance [16,17]. However, Muñoz-Martínez et al. [17] suggest that many professionals lack confidence, training, and structural support to effectively integrate sustainability into dietary counseling, with the availability of updated, sustainability-oriented national FBDG representing one important contextual factor .

A systematic review identified multiple individual and organizational factors influencing the integration of sustainable nutrition into professional practice, including professional knowledge, perceived responsibility, institutional support, and access to practical guidance [18].

These findings suggest that both individual and systemic factors shape the integration of sustainable nutrition into professional practice. However, although FBDG are occasionally mentioned as potential factors or tools in these studies, their specific role and influence have not been explored in depth. Given that health and nutrition professionals in many countries are expected to operationalize FBDG [8], a more focused investigation is warranted. Therefore, the primary objective of this study is to identify factors that influence the implementation of FBDG from the perspective of health and nutrition professionals. A secondary objective is to explore opportunities for strengthening FBDG implementation through targeted support and policy measures.

To explore the multifaceted factors that influence the implementation of FBDG, this study adopts the socioecological framework (SEF), which is a well-established model in public health research [[19], [20], [21]]. The SEF offers a holistic perspective by recognizing that individual behaviors are shaped by, and in turn influence, a complex interplay of personal, social, institutional, and policy-level factors [19,20]. Originally conceptualized by Bronfenbrenner [22] and later adapted by McLeroy et al. [20], the framework identifies five interrelated levels of influence:

1) Intrapersonal factors: individual characteristics such as knowledge, attitudes, behaviors, and self-efficacy.
2) Interpersonal relationships: social networks and support systems, including family, peers, and colleagues.
3) Institutional factors: organizational structures, rules, and workplace norms that shape professional practice.
4) Community factors: connections among institutions and local networks that facilitate or hinder collaboration.
5) Public policy: national and regional laws, regulations, and guidelines that influence professional roles and responsibilities.

In this study, the SEF guided a deductive content analysis [23] of data derived from semistructured interviews and a follow-up quantitative survey. Interviews were conducted with dietitians, nutritionists, physicians, and epidemiologists working across 6 continents (Africa, Asia, Europe, North America, South America, and Oceania) and focused on their experiences with implementing FBDG in their professional contexts. Insights from the interviews were systematically mapped onto the SEF to identify determinants operating at each level. This structured approach enabled a comprehensive understanding of both the personal and contextual factors shaping FBDG implementation in diverse health and nutrition settings [[19], [20], [21]].

## Methods

This study employed an exploratory sequential mixed-methods design [24], integrating qualitative and quantitative approaches to examine the factors influencing the implementation of FBDG among health and nutrition professionals.

### Phase 1: qualitative interviews

In the first phase, semistructured interviews were conducted with health and nutrition professionals, including dietitians, nutritionists, physicians, and epidemiologists. The aim was to explore professionals’ subjective experiences with the implementation of FBDG and to gain in-depth insights into the challenges and enabling factors that influence their successful implementation [25].

Participants were recruited through a combination of convenience sampling [26] at international scientific congresses focused on nutrition, health, and sustainability in Kuala Lumpur, London, and Barcelona, and snowball sampling, which facilitated access to global professional networks. Participants were not selected through a systematic country-based sampling strategy; instead, recruitment sought to maximize geographical diversity by including professionals from as many world regions as possible. During the snowball sampling process, participants were specifically asked to recommend colleagues from different countries and, where possible, different continents to broaden the diversity of perspectives. Although interviews at conferences were conducted in person, those resulting from snowball sampling were held via video call. All interviews were conducted in English, with the exception of three interviews that were held in German.

The semistructured interview guide explored participants’ experiences and views on the use, design, and implementation of FBDG, as well as the role of governmental and institutional stakeholders (Supplemental Material 1). Each interview began with an open-ended introductory question:

“Please tell me why and in which context you have dealt with food-based dietary guidelines in the past.”

Subsequent key questions (typically 5–9 per interview) encouraged participants to elaborate on factors influencing FBDG implementation and expectations for guidelines that promote healthy and sustainable food choices. Examples include:

“What factors influence the implementation of dietary guidelines?”

“When you think about the design of an FBDG, what are your expectations of a guideline that aims to promote healthy, sustainable food choices?”

Follow-up questions were used to elicit further detail or clarification, such as:

“What else is needed to promote healthy, sustainable food choices?”

Each interview concluded with a question inviting recommendations for further participants (“Which other person should I interview?”).

New interviewees were recruited until thematic saturation was reached, which means at a certain point, no additional topics or insights emerged from the data collection [27].

Interview transcripts were analyzed using qualitative content analysis as described by Mayring [23]. The SEF served as a coding structure to categorize responses across five levels of influence: intrapersonal, interpersonal, institutional, community, and policy. Data were first mapped to the corresponding SEF level, then further organized into subthemes to identify patterns and similarities across participants’ perspectives. Coding was conducted independently by two researchers, after which coding decisions were compared and discussed until consensus was reached, thereby enhancing the credibility of the findings.

### Phase 2: quantitative survey

Findings from the qualitative phase informed the development of a quantitative online survey, designed to test and generalize the initial insights. Key themes from the interview analysis were operationalized into Likert-type scale items and multiple-choice questions (Supplementary Material 2). The final questionnaire comprised 43 items structured into five sections:

1) *Professional background*: including participants’ country of residence, education, and job title.
2) *FBDG usage*: for example, “Do you use the national FBDG of the country you are based in for your work (e.g., nutritional counselling or public lectures)?”
3) *Perceived characteristics of FBDG*: for example, “In your opinion, which foods are most underrepresented in FBDG so far?”
4) *Barriers to implementation*: for example, “Please indicate the level of influence of each factor on the implementation of FBDG,” based on statements derived from the qualitative interviews.
5) *Policy and community-level support*: for example, “Which policy or community factors do you consider most important for improving adherence to FBDG? (Select up to three).”

Before full deployment, the questionnaire underwent pilot testing with 14 respondents to assess clarity, relevance, and usability. Feedback from the pilot informed refinements to wording, layout, and item structure.

The final survey was administered over a 3-mo period (1 June, 2024–31 August, 2024). Participants were recruited using convenience sampling. The target population consisted of health and nutrition professionals; however, because no comprehensive international sampling frame exists for this population, probability-based sampling was not feasible. Instead, invitations containing the survey link were disseminated through existing professional networks. International health and nutrition associations were contacted by e-mail and invited to circulate the survey through their member newsletters or mailing lists, with three organizations agreeing to do so. The survey was also shared repeatedly through the authors’ LinkedIn profiles to reach additional professionals working in nutrition and public health. Participation was voluntary and anonymous. Quantitative data were analyzed using Python 3.12.7.

### Ethical approval

The protocol for the study was reviewed and cleared by the University of Goettingen Research Ethics Committee on 13 July, 2023, for the qualitative part of the study, and on 28 February, 2024, for the quantitative part of the study. All participants gave informed consent before taking part.

## Results

This section presents the study findings, first describing the composition and demographics of the sample, and then reporting the qualitative insights from expert interviews alongside the quantitative results from the online survey.

### Participants

A total of 20 participants were interviewed during the first phase of this study. This group was predominantly composed of dietitians (*n* = 9) and medical doctors/physicians (*n* = 8). The remaining participants included one epidemiologist, one pharmacist, and one nutritionist. Participants were based across six continents—Africa, Asia, Europe, North America, South America, and Oceania—reflecting a broad geographical diversity.

The second phase of the study involved an initial recruitment of 120 participants. After data cleaning, two responses were excluded because of implausibly rapid questionnaire completion times, yielding a final dataset of 118 participants.

Participants were able to indicate multiple areas of profession and education. Consequently, the aggregated percentages presented in Table 1 will sum to >100%. For classification, any participant who selected dietitian as at least one of their professions was categorized as such, noting that this may represent a combination with other roles. An analogous method was applied to the remaining professional categories.

**TABLE 1:** Demographic information of the 118 participants in phase 2.

Demographic variables	*n* = 118	100%
**Professions (multiple choice)**		
Nutritionist or nutritionist in combination with other professions	48	40.7%
Dietician or dietician in combination with other professions	42	35.6%
Physician or physician in combination with other professions	14	11.9%
Researcher or researcher in combination with other professions	16	13.6%
Nurse or nurse in combination with other professions	8	6.8%
Other (e.g., teacher, epidemiologist, governmental official) or other in combination with other professions	34	28.8%
**Type of organization working for**		
Self-employed	25	21.2%
Hospital, clinic, or medical center	21	17.8%
Nongovernmental organization (e.g., nonprofit, foundation)	19	16.1%
Private industry	15	12.7%
Academia	11	9.3%
Government	11	9.3%
Education	6	5.1%
Health insurance company	1	0.8%
National nutrition society	1	0.8%
**Dietary concepts or nutrition philosophies (multiple choice)**		
Whole food plant-based diet	64	54.2%
Mediterranean diet	50	42.4%
Vegan diet	43	36.4%
Planetary health diet	29	24.6%
Flexitarian diet	25	21.2%
Vegetarian diet	20	16.9%
Low-carbohydrate diet	19	16.1%
Clean eating	16	13.6%
Intermittent fasting	14	11.9%
Low-fat diet	14	11.9%
DASH diet (dietary approaches to stop hypertension)	13	11.0%
Religious-based diets (e.g., Buddhist, Christian, Hindu, Islamic, Jain, Jewish, etc.)	9	7.6%
Not aligned with any diet/philosophy	7	5.9%
Other (e.g., ayurvedic, paleo, raw food diet)	15	12.6%

Region	Countries included	Respondents *n* = 118
Africa		14.16%
Northern Africa	Egypt	1
Sub-Saharan Africa	Ethiopia, Gambia, Ghana, Nigeria, South Africa	11
Asia		20.06%
South-eastern Asia	Malaysia, Indonesia, Singapore	8
Southern Asia	Bangladesh, India, Sri Lanka	5
Western Asia	Israel, Lebanon, Saudi Arabia, United Arab Emirates	4
Oceania		25.96%
Australia and New Zealand	Australia, New Zealand	21
Melanesia	Fiji	1
Europe		44.07%
Eastern Europe	Czechia	1
Northern Europe	Finland, Ireland, Sweden, United Kingdom	19
Southern Europe	Italy, Portugal, Spain	26
Western Europe	Austria, France, Germany, Switzerland	6
Northern America	Canada, United States of America	9 (7.63%)
Latin America and the Caribbean	Argentina, Chile, Colombia, Peru	6 (5.08%)

Participant countries of residence were organized based on the FAO world regions classification. This approach was adopted to illustrate the global perspective of the study and should not be interpreted as an assumption of homogeneity among nutrition and health professionals within the designated regions.

### Qualitative results

**FIGURE 1:**
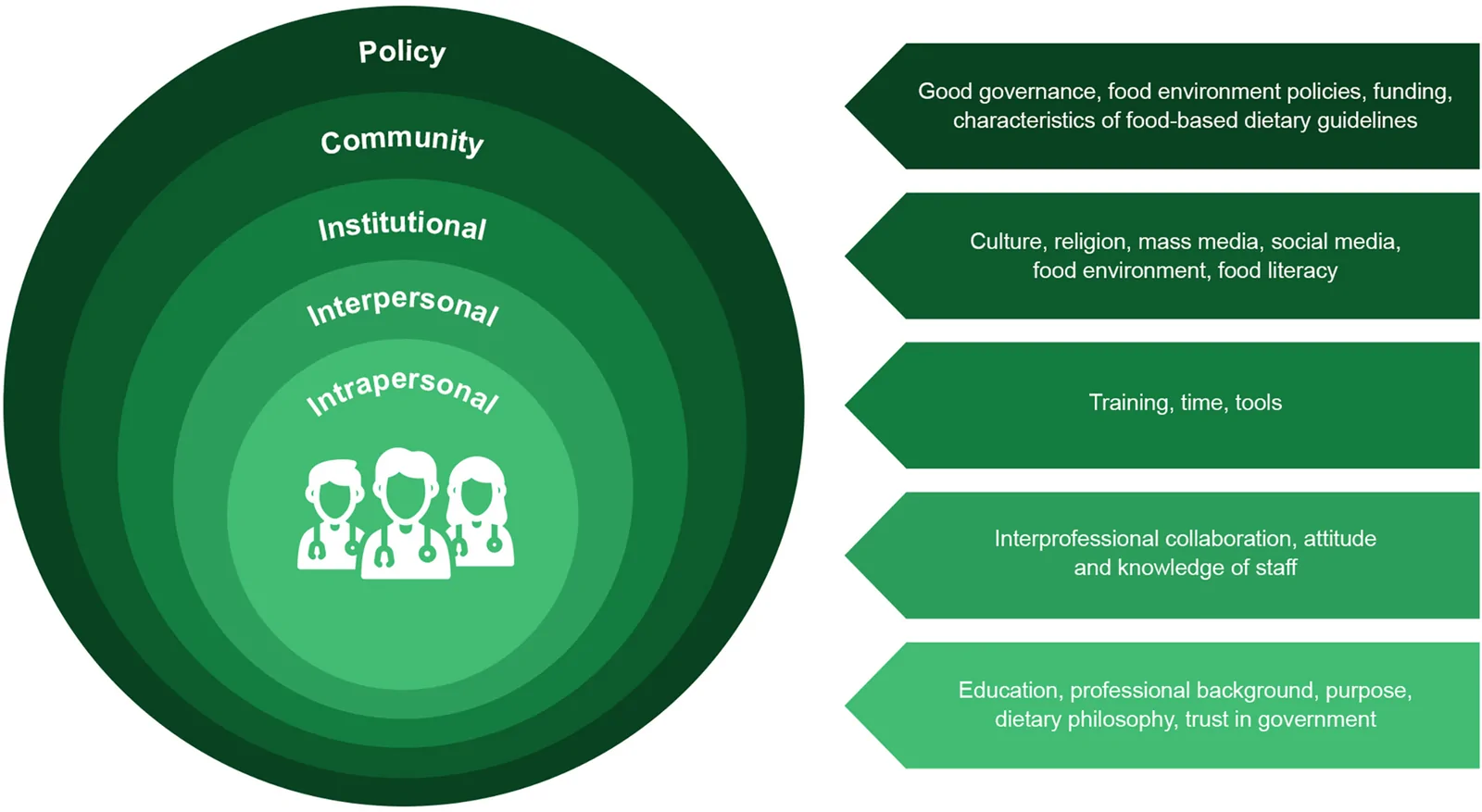
Socioecological framework of factors influencing the implementation of food-based dietary guidelines by health and nutrition professionals.

A description of factors influencing the implementation of FBDG by health and nutrition professionals is shown in Figure 1. These factors are structured into five levels of influence and used as a categorization system for the qualitative results.

The following section describes and summarizes the results of the qualitative interviews together with illustrative quotes.

#### Intrapersonal

##### Educational and professional background

FBDG appeared more relevant to participants with formal nutrition training (dietitians and nutritionists) than to physicians, who consistently described receiving little nutrition education during medical training.

**Dietician Malaysia #2**

*When I started practicing as a nutritionist, working with patients and clients, of course, that is one of the guidelines that I need to have because I don't want to give any advice that is not evidence backed.*

**Physician US**

*The only thing that I learned in four years of medical school about nutrition was one hour, mainly on supplements and artificial feedings for patients that are in the hospital. As far as the dietary guidelines, there was maybe 10 s mentioned that there's dietary guidelines that are released that's done by the government, and you can use that as a guide. And that was it.*

##### Purpose and dietary philosophy

Participants preferred FBDG that extended beyond healthy eating to include environmental sustainability and cultural, ethical, and religious considerations. Several professionals also reported using alternative frameworks, such as the EAT-Lancet diet or the Canadian FBDG, when these better aligned with their own dietary philosophy.

**Physician Netherlands:**

*I never use the official plate. I always use the EAT-Lancet [planetary health diet]*

**Dietician Portugal:**

*Well, honestly, I have my own. Yeah, it's a plate. (...) And I created it based on the Portuguese, the Canadian and I did some research and blended with my experience and created my own.*

**Dietician South Africa #1:**

*(...) it [environmental sustainability] should absolutely be included there [FBDGs] because it slots in with food availability, acceptability and accessibility.*

##### Trust in government

Trust in government emerged as an important factor influencing the implementation of FBDG. Although most participants expressed confidence in the scientific committees or nutrition societies responsible for developing the evidence base, they were less confident in the governments’ role in the final decision-making process. Concerns centered on perceived political and industry influence over the final recommendations and on infrequent updates of the national FBDG. Participants suggested that these factors reduced confidence in the guidelines and, consequently, their willingness to use them in practice.

**Medical doctor Chile:**

*In Chile, some powerful food industries such as the dairy consortium are involved in the building of the guidelines, which undermines public and health professionals' trust in them given their private conflicts of interest.*

**Dietician USA #2:**

*(...) having worked for the USDA [United States Department of Agriculture] myself, I know the bureaucracy that goes along with it. And, you know, I think politics comes into play in a lot of the higher ups. So, I kind of have a feeling that there are some constraints about what they can and cannot say when it comes down to the end of the day.*

**Dietician Portugal:**

*I would like to trust (...). They are trying their best, I think. I totally think so. But the livestock lobby, the meat lobby and the pressure here in Portugal, it's huge.*

#### Interpersonal

##### Attitude and knowledge of staff

On the interpersonal level, it was found that the attitude and knowledge of the staff with whom the nutrition and health professionals work can be factors influencing the implementation of the FBDG. In particular, the lack of nutritional knowledge among doctors was criticized—this criticism came from all interviewees, including the medical doctors themselves. In addition, the role model function of healthcare staff was seen as an important supporting factor for patients. In other words, to what extent the staff themselves follow the FBDG and consider the recommendations to be useful.

**Medical doctor India:**

*People go to the doctor when they have an illness. They believe their doctors more than any other person when it comes to health and the doctors doesn't know what is the right nutrition. That is a challenge, which we face most cases.*

**Medical doctor Chile:**

*Physicians in Chile are not well educated in nutrition, especially in its importance for long term health. This leads to a lot of misunderstandings, and underestimation of its importance on the health of the patients and population.*

##### Interprofessional collaboration

Participants highlighted interprofessional collaboration as an important facilitator of FBDG implementation. Dietitians frequently reported that their expertise was underrecognized by physicians, leading to underutilization of specialist nutrition knowledge. Improved collaboration and referral between healthcare professionals were considered essential for effective implementation.

**Nutritionist Fiji:**

*I don't know if that's the best word, but there's a bit of arrogance as well from health professionals, especially doctors and people who are not qualified in dietetics, meaning they think they know already, even though they never really did any studies. Or if they did, it was 30 years ago.*

**Dietician USA #1:**

*(...) a lot of physicians really don't understand what dietitians do. They don't realize how helpful we can be. (...) It really has been a problem for decades that registered dietitians are underutilized and underappreciated. So maybe having the dietary guidelines really reflecting like, hey, you may not know all of these details. Don't worry, we're not expecting you to be experts (...). Send your patient to a dietitian.*

**Dietician Malaysia #1:**

*We should get trained to convey the message (...). And it's like a competition of who says better. It should be a collaboration and it should also be consistent.*

#### Institutional

##### Training

Institutional provision of nutrition training emerged as an important factor influencing FBDG implementation. Although medical doctors are often the first point of contact for health and nutrition-related concerns, physicians consistently reported receiving little formal nutrition education and limited training on FBDG, whereas dietitians described FBDG as a core component of their professional education.

**Physician Germany:**

*We don't have a nutrition course in medical school. There was one optional course that I took, but it wasn't good, and I can remember maybe hearing the word DGE [German Nutrition Society], for example, but I can't remember gaining any substantial knowledge about healthy eating during my studies.*

**Dietician South Africa #1**

*It [the FBDG] was a big part of our training.*

**Dietician Portugal:**

*Here in Portugal, they are very proud of their food wheel [graphical representation of the FBDG]. At the university we had a lot of classes about the food wheel and the dietary guideline.*

These qualitative findings suggest that limited institutional training may act as a barrier to FBDG implementation. This was supported by the survey, where respondents who had studied FBDG in detail during their professional education were more likely to report using them in practice (Table 2).

**TABLE 2:** Reported use of food-based dietary guidelines (FBDG) by level of curricular exposure to FBDG (*n* = 118).

FBDG used in work	No	Sometimes	Yes
FBDG included in curriculum			
I cannot remember	4%	2%	3%
No	3%	11%	8%
Yes, but they were only mentioned briefly	8%	14%	8%
Yes, they were discussed in detail	5%	9%	25%

##### Time and tools

Limited consultation time and insufficient implementation resources were perceived as important barriers, whereas practical materials such as graphics, brochures, and patient handouts were viewed as valuable implementation tools.

##### Medical doctor Chile:

*In Chile, doctors don't have a lot of time when attending people. When I do my independent consultations, I use one hour per patient. In the public system, we usually have 15-30 minutes per patient, and a lot of other issues and paperwork to be resolved, leaving little to no time for nutrition counseling.*

**Physician Germany:**

*One of the factors is probably what material is available that I can use. So are there good graphics that I can simply incorporate into the situation with the patient, are there flyers that I can order [from the responsible authorities]?*

#### Community

##### Culture and religion

Participants emphasized that FBDG should be sufficiently flexible to accommodate cultural, religious, and ethnic diversity, enabling recommendations to be adapted to different dietary traditions.

**Dietician South Africa #2:**

*In South Africa, for example, we have different cultures, a lot of different cultures. And so not everyone eats the same foods, not everyone understands the same things. There's a lot of factors to take into consideration, and I think that's important to really make things adaptable, like understanding that it might need to be adapted based on those factors.*

**Dietician USA #2:**

*You know, I have referred to it [national dietary guideline] in the past, but I don't normally use it because it has a dairy icon on it. And a lot of patients, especially low income, are lactose intolerant, and it's not part of their culture.*

**Dietician Malaysia #2:**

*Malaysia is a multiracial country, right? (...) I find it a bit hard when I want to implement with patients because, you know, sometimes patients would be like, okay, I'm Chinese, how can I actually use this guideline into my daily life? (...) So understanding the culture, where they came from, is important.*

##### Mass media and social media

Social media was viewed as both a barrier and an opportunity. Participants highlighted widespread nutrition misinformation while advocating for stronger government communication campaigns, including the use of social media and influencers.

**Dietician Portugal:**

*I think, the misinformation in social media today, it's a very big issue that influences a lot. I can't even count how many patients are very afraid of carb sources and everything. So the media and especially the social media, demonizing carb sources (...).*

**Dietician USA #1:**

*Most Americans are like, “what's that? I've never heard of that, I don't care, it's a government website. It's awful.” (...) we should have some TikTok influencers talking about the dietary guidelines in a way that's fun, right?*

##### Food literacy

Participants emphasized the importance of improving food literacy through nutrition education, particularly by integrating nutrition into school curricula and public education initiatives. However, they also stressed that education alone is insufficient to achieve meaningful dietary change.

**Medical doctor Chile:**

*So I think everything is related. Everything, the education of people, that children have in their schools, the offer of foods that restaurants have, the offer of foods that institutional canteens have, not to mention the children's ones, the kindergartens, how we celebrate culturally, like how we are celebrating birthdays (...).*

##### Food environment

Participants identified community food environments as a major influence on FBDG implementation. Retailers, restaurants, community catering services, the food industry, and other community actors were viewed as having an important role in making healthy and sustainable food choices easier. Several participants highlighted that commercial food environments could either support or hinder implementation through product placement, marketing, and food availability.

**Dietician South Africa #1:**

*We have retailers (...). They [the retailers themselves] implemented a policy for a healthier checkout aisle. And they don't put any, like, sweets or whatever they classify as unhealthy in the checkout aisle because they understand the checkout aisle is where individuals are vulnerable. They tired, they're waiting in line, they're bored. Then they start filling their shopping trolley with things that they wouldn't necessarily have bought otherwise.*

**Epidemiologists The Gambia:**

*Depending on the context, different things might needed and in our context, I think is largely about getting people to consume more fruits and vegetables and making the environment in such a way that these are there and changing some bit of cultural practices here.*

**Dietician USA #2:**

*It's not demonizing industry. I mean they're in it for making money, but they could be more responsible and helpful in those ways. (...) They're mega-corporations they own most of the food system. They can make a difference by what they sell, what they say and what they advertise.*

#### Policy

##### Characteristics of FBDG

Participants described several characteristics of FBDG that influence their implementation. These findings are presented under three related subthemes: content-wise, visual representation, and a dual-format strategy for professionals and the public.

##### Characteristics of FBDG: content-wise

Participants consistently highlighted that the content of FBDG determines their implementation. They emphasized the importance of regular updates to reflect the latest scientific evidence, inclusion of local and indigenous foods, greater representation of plant-based options, and practical advice on affordability.

**Dietician Malaysia #2:**

*Here in Malaysia, they always have this one fundamental question of can we afford it? So I think a bit of information about costs in the guideline would be great.*

**Dietician The Gambia:**

*(...) when you do food consumption surveys, you do dietary record, you ask people, have you eaten fruits and vegetables? They think of these exotic fruits and vegetables whilst we have, you know, the indigenous fruits and vegetables that people don't even recognize as fruits and vegetables. So you could see in the calabash [food graphic in The Gambia] that we had, people could recognize their local fruits that you would not find in the developed world.*

**Dietician Portugal:**

*I think that to show plant-based options is a very important tool and most of them [FBDGs] are not doing it, right? So I think the Netherlands and Canada are very good in this point to including plant-based products to show that you can choose it (...). But if it's not in the dietary guidelines, probably you will not recommend it for your patients (...). So I think to include more plant-based options and to show how to choose the good ones. I think this is a point that the national dietary guidelines need to improve.*

**Dietician USA #1**:

*We don't need a dairy group. We're really we're talking about calcium here. Let's have a calcium group then, or a list of calcium-rich foods and push everything else either in the protein group (...). Most people get plenty of protein. You know, there's some subgroups that need it. Um, but calcium is really an issue (...). They could talk about the bioavailability of certain green leafy vegetables, you know, kale and all of that. Give that information. (...) They only list dairy and fortified soy milk because they say everything must mirror dairy. And I'm like, well, we don't do that for any other group. We don't say you have to have the same amount of vitamin C as an orange to be a fruit, right? You don't do that for anything else.*

**Nutritionist Germany:**

*I think they should really be more flexible in terms of ethical and religious aspects, which is becoming increasingly important nowadays. For example, nobody who eats a fully plant-based diet [vegan diet] feels represented by the German dietary recommendations.*

**FIGURE 2:**
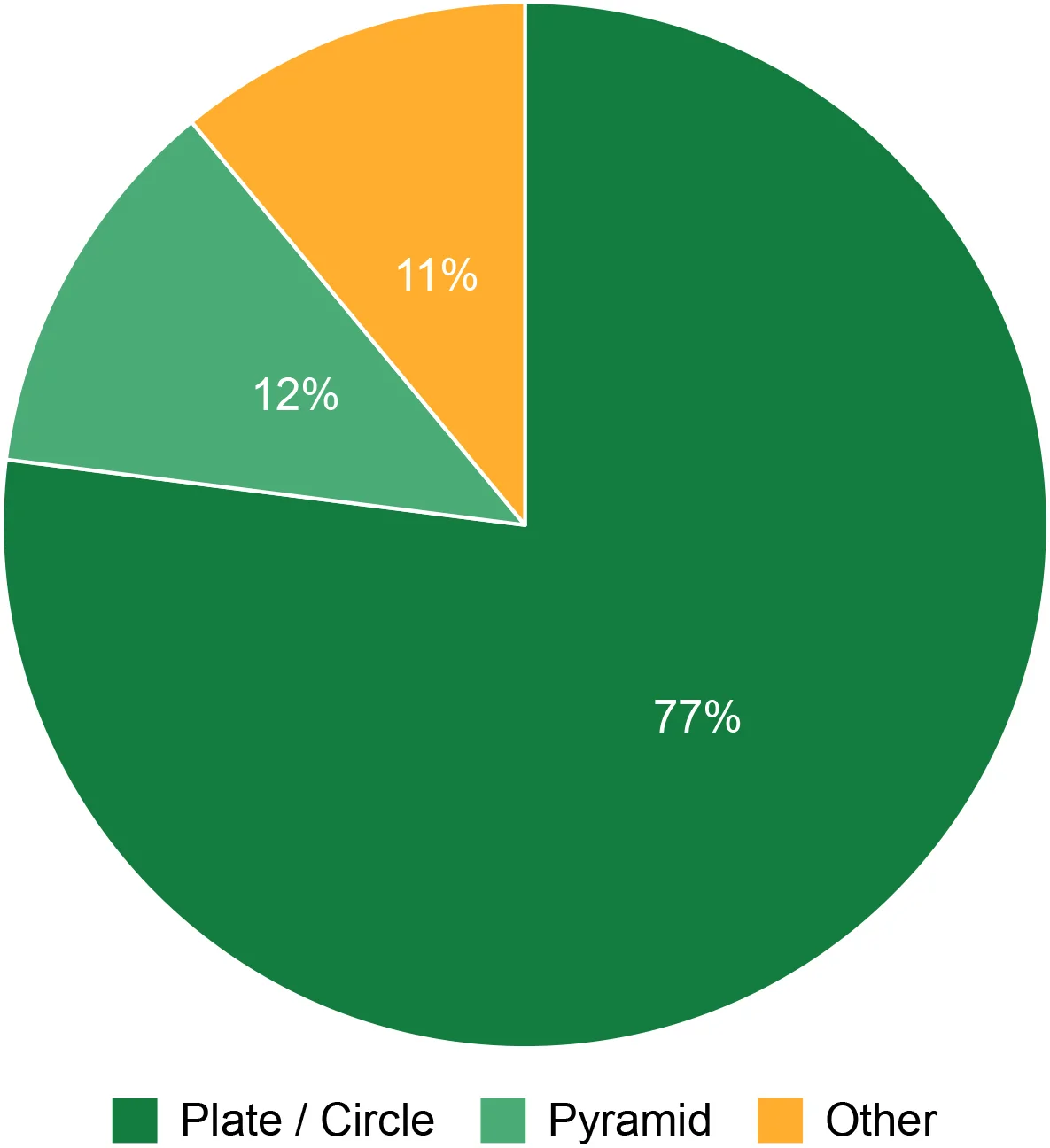
Preferred shapes of national FBDG food graphics among health and nutrition professionals (n = 118). FBDG, food-based dietary guidelines.

These qualitative findings informed several survey questions on FBDG content. Consistent with the interviews, survey respondents most frequently identified legumes, plant-based milk alternatives, and nuts and seeds as underrepresented food groups (Figure 2).

##### Characteristics of FBDG: visual representations

Visual representations, such as plates, pyramids, or other diagrammatic formats, were considered essential for communicating FBDG, with most participants preferring plate-based graphics over pyramids because they were perceived as more intuitive and easier to use in practice.

**Nutritionist Fiji:**

*The pyramid, the plate and the rainbows and things like that. So some can be more confusing and some can be quite useful (...). My favorite one is the plate, because you can really use it in your awareness work and say, you know, that's how your plate should look.*

**Dietician Malaysia #2:**

*Because I don't use the pyramid per se. When I talk to my patients, I only use the plate because I feel that is the most relatable to the patient. It's much easier to explain.*

**Epidemiologists The Gambia:**

*We have two. We had a basket and then we had a calabash. Because this is what people identify with food. And there was a lot of discussion and debate around which one is more appropriate (...). I think different populations have different literacy levels. And it makes communication easier. (...) So I think these [graphical elements] are still very, very useful parts of food-based dietary guidelines.*

**Medical doctor Chile:**

*Last time it was a circle (...). And now it's not like a plate. It's not like a pyramid, but it's just a number of messages (...). I think it's better to have a graphic.*

##### Characteristics of FBDG: dual-format strategy

When asked about the potential value of having two versions of FBDG—one simplified for the general public and another more detailed version for professionals—nearly all interviewees indicated that they would find this approach highly beneficial. Support for this idea was also evident in the survey, where 84.8% of respondents agreed that FBDG should be available in separate versions for professionals and the general public.

**Dietician Malaysia #1**:

*How easy is it to understand for the public? So personally I think it should be two versions, because for us we want to know more.*

**Epidemiologists The Gambia:**

*Yeah, we've not really thought of that but I think it's a good idea. So we can have a more simplified version for the public and we have more details, maybe a facilitator manual (...). This would be really very, very useful because sometimes we are not able to add more information because we think it will be too much for the general public. And this is too much nutrition information (...). And sometimes when you have young professionals coming out, they really need some background information, even though they have just graduated. So if you have the one for health professionals and one for the general public, that will be perfect.*

##### Good governance

According to the United Nations, good governance is defined as “participatory, consensus oriented, accountable, transparent, responsive, effective and efficient, equitable and inclusive, and follows the rule of law. Furthermore, it seeks to ensure that corruption is minimized, the views of minorities are taken into account and that the voices of the most vulnerable in society are heard in decision-making. It is also responsive both to the present and future needs of society.” [28]. One or more of these aspects were mentioned in almost all interviews as important for the development and implementation of FBDG. These qualitative findings were strongly supported by the survey, in which respondents consistently rated key principles of good governance—including transparency, the use of current scientific evidence, and the prevention of corruption—as highly important.

**Medical Doctor Germany #2:**

*So I thought it was good that the public was involved in the development of the new guidelines (...). And a very important aspect is transparency in many areas. That they are independent of economic and political interests, these are very important aspects and, above all, that [the FBDG] is regularly updated (...). I think the whole process needs to be more streamlined, more regular and more flexible.*

**Physician US:**

*I think the positive part of the process is that people are allowed to comment on them [the american FBDG] at multiple stages and that it is announced and it is fairly open (...).*

It should be noted that the interview with the United States physician was conducted in September 2023. The 2025–2030 United States Dietary Guidelines, unveiled in January 2026, have faced significant criticism for allegedly ignoring public consultation and scientific advice.

**Medical doctor Ireland:**

*But what we see, what our political leaders do, they seem to torpedo anything which is a threat to the dairy cattle industry (...). And so in Brussels, with most recent agricultural laws that have been played out now with the nitrate limits. Our dairy farmers [in Ireland] have the highest nitrate limits in any European country and are still being extended.*

**Dietician The Gambia**:

*You [the government] need to have a monitoring framework.*

##### Food environment policy

Participants viewed government regulation of the food environment as essential for FBDG implementation, highlighting procurement policies, subsidies, taxation, front-of-pack labeling, and mandatory nutrition standards as key policy levers. Similarly, the survey findings supported these qualitative insights, with respondents ranking these government regulations among the most important policy measures to improve FBDG implementation (Figure 7).

**Physician Germany**:

*I think the most important thing is to shape the food environment and, within that, community catering, so that there are very clear subsidies, incentives, labelling, perhaps also mandatory standards [based on the national dietary guidelines].*

**Dietician USA #1**:

*Food companies spend millions and millions of dollars on advertisements. How about if they get federal reimbursement and subsidies that they have to spend a certain amount of that advertising budget on promoting things that are kind of connected to the dietary guidelines?*

**Dietician South Africa #2**:

*There are some initiatives, like school feeding programs and things like that. Recently, they created the sugary beverage tax.*

**Dietician Portugal**:

*(...) the front-of-pack-label works very well here in Portugal. There are some reports, some research in this area. And it really influences people to choose better options.*

##### Funding.

Insufficient funding was consistently identified as a major barrier to the development, revision, and implementation of FBDG, with participants also recognizing an important supporting role for international organizations such as the FAO [29].

**Epidemiologists The Gambia**:

*Funding is an important part of it (...). So I think what influences whether this [the FBDG] will be implemented or not largely is* *funding and also if it is integrated within the health system (...).*

**Dietician South Africa #2**:

*I feel like it's the dietitians trying to advocate and trying to be heard. That's why I would say from a government perspective, there's very little* *support. And it's seen in like lack of* *funding when it comes to nutrition.*

**Dietician USA #2**:

*(...) it comes down to* *funding a lot of the times in terms of implementation.*

### Quantitative results

A total of 120 questionnaires were received. After data cleaning, two responses were excluded because of implausibly short completion times, resulting in a final sample of 118 participants. Overall, 69.5% of surveyed health and nutrition professionals reported that FBDG had been included in their formal curriculum (e.g., university, medical school). However, within this group, 30.5% indicated that FBDG were only mentioned briefly.

In professional practice, the majority of respondents (80.5%) reported using the national FBDG of the country in which they are based, for example, in nutritional counseling with clients or in public lectures. When analyzed by professional background, no clear differences in the use of FBDG were observed. However, respondents who had studied FBDG in detail during their curriculum were more likely to report applying them in practice (see Table 2).

When asked about the usefulness of their national FBDG in promoting healthy and sustainable food choices, most professionals rated the guidelines as only somewhat or slightly helpful (57.6%). A small proportion considered them very helpful (7.6%), whereas 14.4% stated they were not helpful at all.

Additional subgroup analyses comparing respondents from high-income countries (HICs) (*n* = 89) and low- and middle-income countries (LMICs) (*n* = 29) revealed largely similar perceptions across most survey items. The only statistically significant difference concerned the perceived usefulness of the national FBDG. Participants from LMICs rated their national FBDG as significantly more helpful in promoting healthy and sustainable food choices than participants from HICs (LMIC: mean = 2.57, SD = 1.14 compared with HIC: mean = 3.39, SD = 1.08; *P* = 0.002). No other consistent differences between the groups were identified.

Expectations for FBDG that promote healthy and sustainable food choices were also explored (Table 3). The most frequently cited priorities were that guidelines should be based on the latest scientific evidence (61.0%), clearly indicate which food choices are optimal for health (57.6%), include a strong recommendation to increase plant-based foods (55.1%), and be adaptable to local contexts (52.5%).

**TABLE 3:** Key expectations of an FBDG promoting healthy and sustainable choices.

Key expectations of an FBDG promoting healthy & sustainable choices	
It should…	% (*n* = 118) (multiple choice)
... be based on the latest scientific evidence.	61.01%
... tell people which food choices are optimal for health.	57.63%
... include a clear message to eat more plant-based foods.	55.08%
... be possible to adapt the recommendations to the local context.	52.54%
... include food that is available to the majority of the citizens.	50.83%
... give recommendations on what will either improve, manage, or prevent diseases.	48.31%
... include a clear message to reduce the consumption of meat.	48.31%
... be flexible and inclusive.	46.61%
... inform people about which food groups have the least impact on the environment.	44.07%
... reflect the barriers people face when choosing healthy, sustainable foods.	42.37%
... break information down with infographics.	39.83%
... include recommendations on how to plan a vegetarian and vegan diet well.	38.98%
... include recommendations on the food-system level.	33.9%
... include myth-busting.	33.05%
... be applicable to clinical practice.	31.36%
Everything is equally important.	22.03%

Abbreviation: FBDG, food-based dietary guidelines.

When asked which expectations are currently not being met, the most common responses were the provision of information on healthy, sustainable diets on a budget (47.5%) and the integration of behavioral change strategies (47.5%). This was followed by inclusion of ecological sustainability (42.4%) and recommendations adapted to cultural aspects (41.5%).

With regard to the preferred shape of the official FBDG graphic, qualitative interviews indicated a clear preference for the plate format, which was confirmed in the quantitative survey. Overall, 77.1% of respondents selected the plate/circle shape as their favorite, compared with 11.9% who preferred the pyramid and 11.0% who chose other shapes (e.g., rainbow, pagoda, fruit; summarized as “other”) (Figure 2). Reported reasons for preferring a given shape included ease of understanding (35.6%), clarity about which foods should be consumed more compared with less often (32.2%), and perceived relevance to people’s reality (29.7%).

**FIGURE 3:**
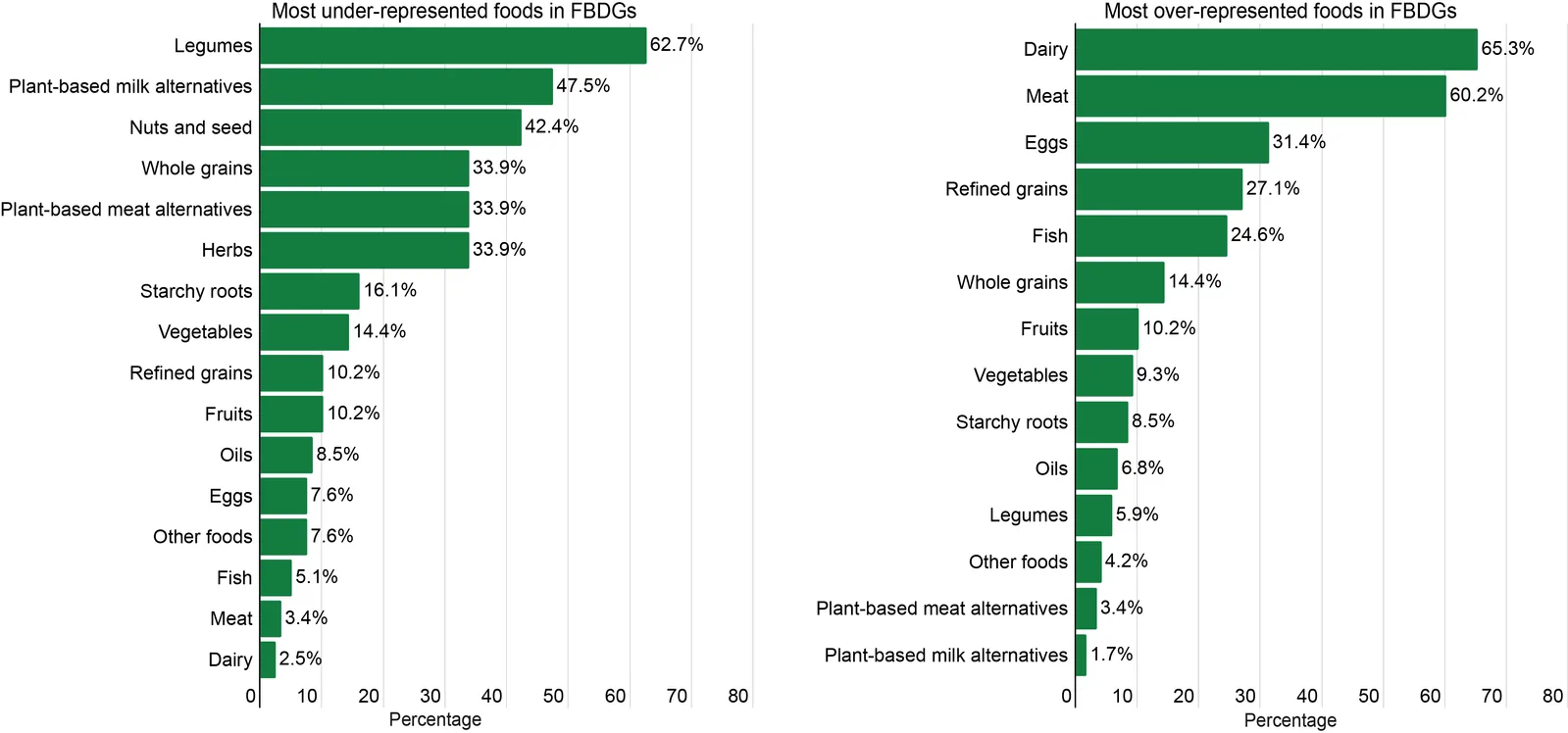
Nutrition and Health Professionals’ Views on Under- and Overrepresented Foods in FBDGs. n=118.

The participating nutrition and health professionals were also asked to identify foods they considered underrepresented and overrepresented in the current FBDG (Figure 3). The most frequently cited underrepresented foods were legumes (62.7%), plant-based milk (47.5%), and nuts and seeds (42.4%). Conversely, dairy (65.3%), meat (60.2%), and eggs (31.4%) were most often identified as overrepresented.

**FIGURE 4:**
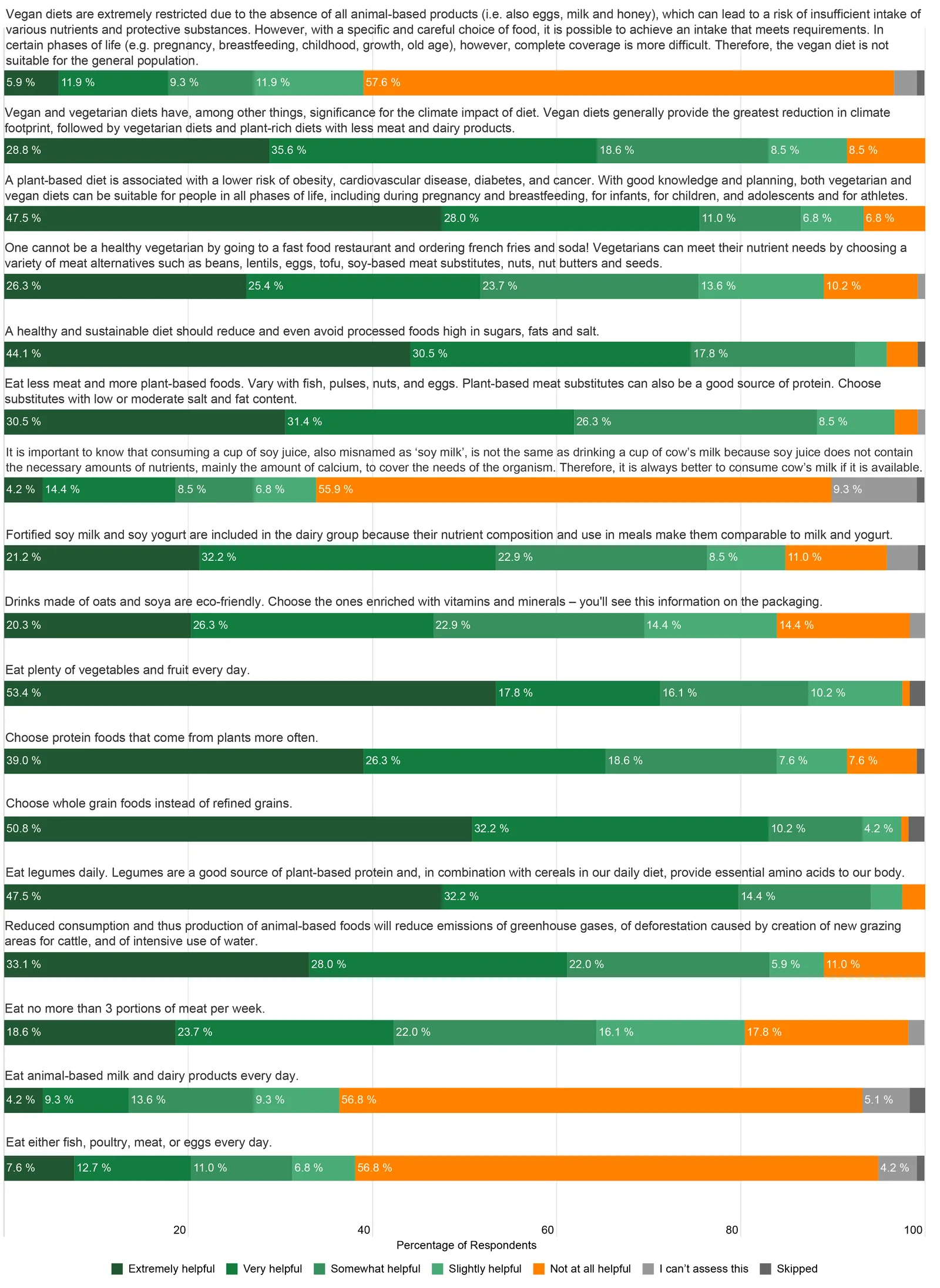
Perceived Helpfulness of FBDG Statements for Promoting Healthy and Sustainable Food Choices.

To further explore the role of specific recommendations, participants rated paraphrased statements from existing FBDG according to their perceived usefulness for promoting healthy and sustainable food choices (Figure 4).

**FIGURE 5:**
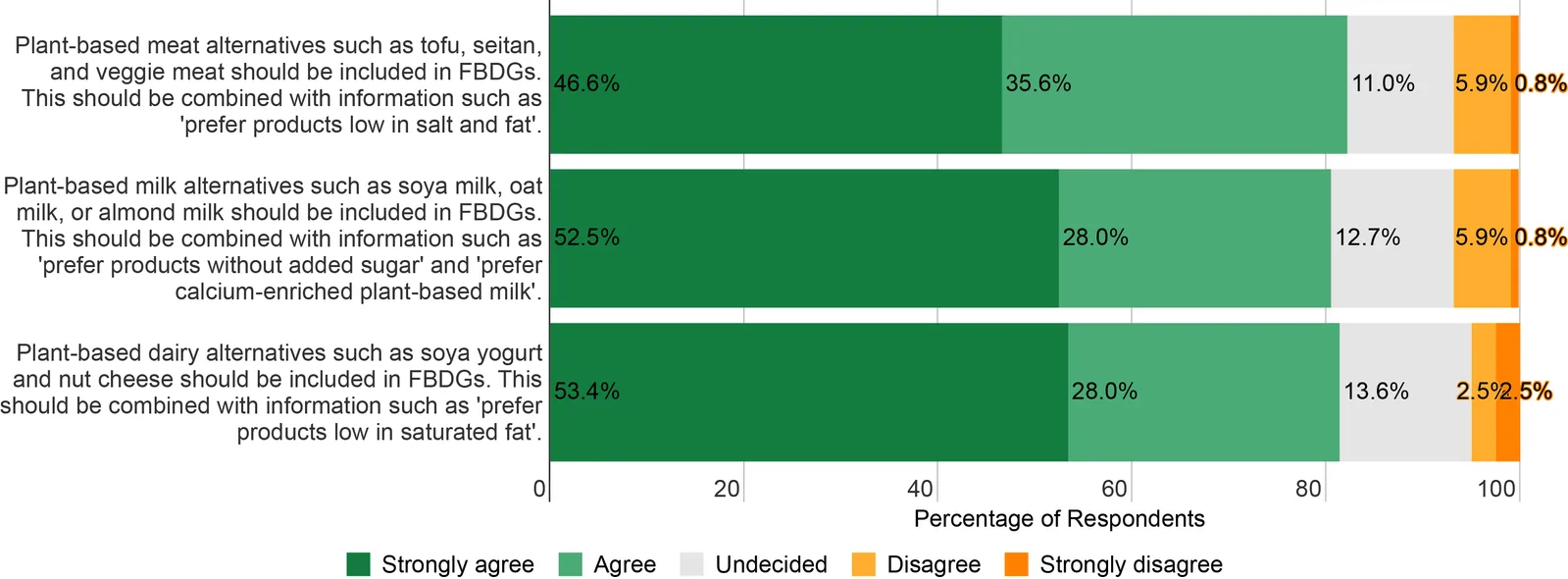
Attitudes of Health and Nutrition Professionals on the Inclusion of Plant-Based Alternatives in FBDGs.

In addition, given the growing public interest in plant-based alternatives and the expanding scientific evidence base on their benefits and limitations, participants were asked whether these products should be explicitly included in FBDG. A large majority strongly agreed or agreed with the inclusion of plant-based meat alternatives such as tofu, seitan, and veggie meats (81.4%), plant-based milk alternatives such as soy, oat, or almond milk (80.5%), and plant-based dairy alternatives such as soy yogurt or nut-based cheeses (82.2%) (Figure 5).

Although the primary target group of FBDG is the general population, recommendations can extend beyond this group. When asked whether FBDG should aim to be inclusive and diverse, the vast majority of respondents (85.6%) strongly agreed or agreed that guidelines should balance ethical, ecological, religious, and economic aspects that influence food choices. Furthermore, 90.8% strongly agreed or agreed that FBDG should provide information for well-planned vegetarian and vegan diets.

Participants were also asked how helpful they would find a dual-format FBDG system—one easy-to-understand version for the general public and a more detailed version for professionals. The majority considered this extremely or very helpful (84.8%), whereas only 6.8% indicated it would be slightly or not at all helpful. This strong endorsement confirms the findings from the qualitative interviews.

Regarding professional training, 45.8% of respondents reported that they were unaware of any FBDG training offered by the responsible authority (e.g., Ministry of Health or Nutrition Society) in their country, and 33.1% were unsure. Only 21.2% indicated that such training was available. Nevertheless, interest in formal training was high, with 83.9% stating that they would participate if it were officially offered.

Participants rated the importance of various aspects to ensure good governance in FBDG development. Across the sample, all aspects identified in the qualitative phase were consistently rated as important. In particular, transparency in the FBDG development and revision process was rated as extremely or very important by 99.1% of respondents (0.8% not important at all). Similarly, informing decision-makers about the latest scientific evidence was considered extremely or very important by 95.8% of participants, and preventing and combating corruption was rated as extremely or very important by 84.7%. Adaptability to nutrition and food trends was rated as relatively less important in comparison.

**FIGURE 6:**
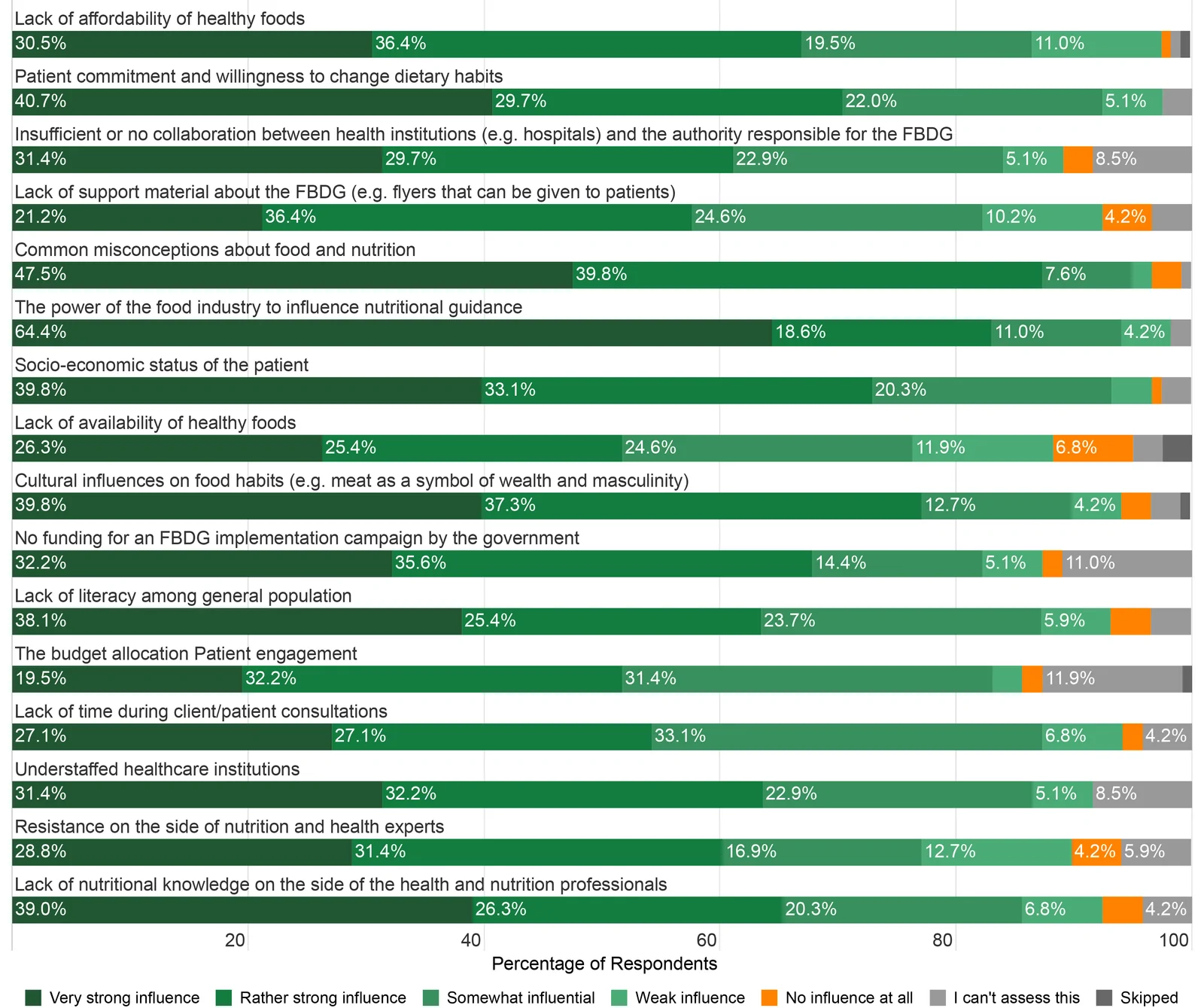
Health and nutrition professionals’ views on barriers to implementing the FBDG. n=118.

Health and nutrition professionals also assessed the influence of various barriers and factors on FBDG implementation (Figure 6). These barriers and factors were initially identified in phase 1 of the study. The most influential factor was the power of the food industry (83.0% very or rather strong influence, including 64.4% rating it as very influential), followed by common misconceptions about food and nutrition (87.3% very or rather strong influence, including 47.5% very strong influence) and cultural influences on food habits (77.1% very or rather strong influence). Conversely, factors such as patient budget allocation and lack of support materials (e.g., informational flyers) were perceived as less influential.

**FIGURE 7:**
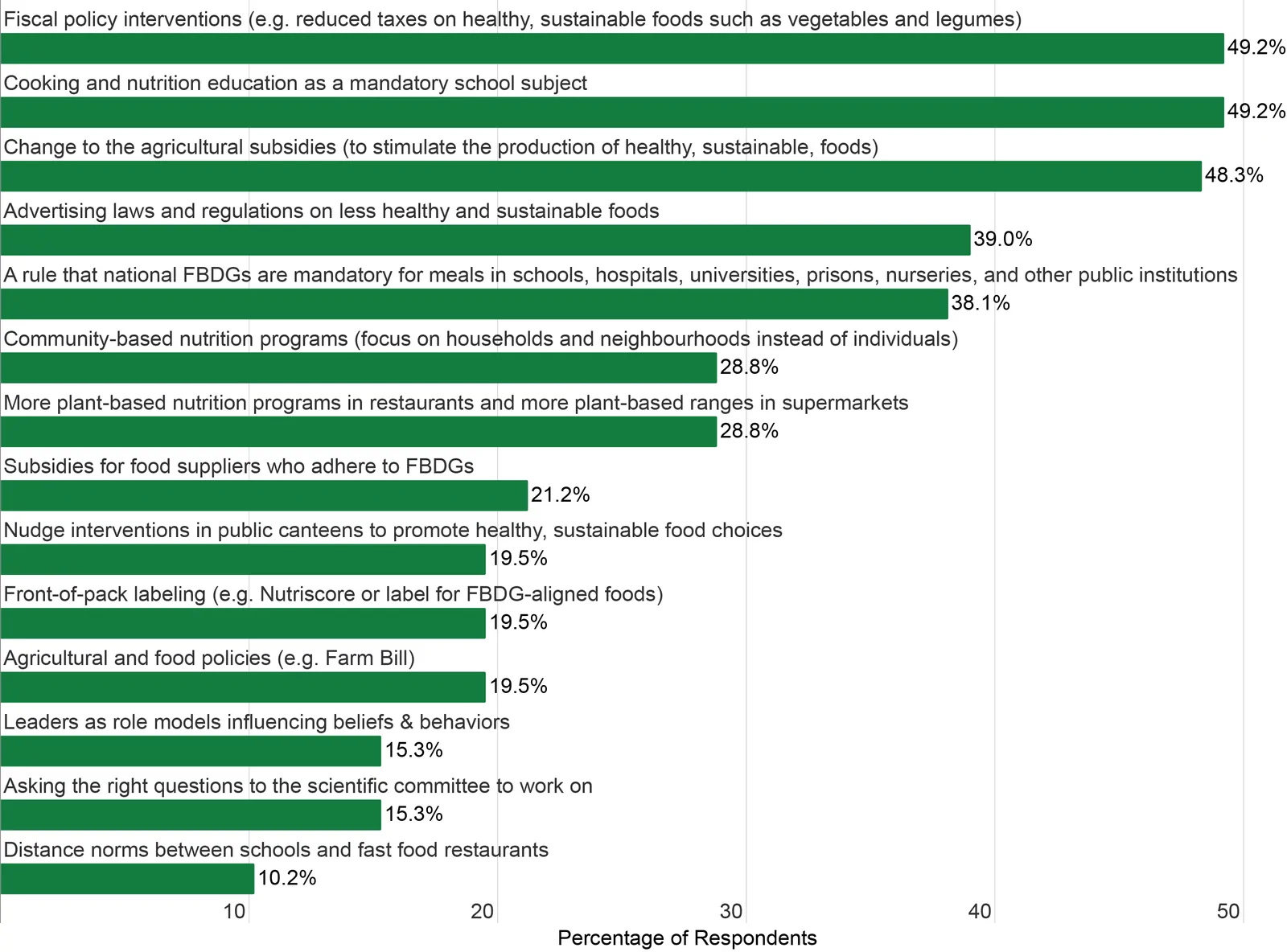
Key Policy and Community Factors Affecting Adherence to FBDGs.

Regarding policy and community factors affecting adherence to FBDG, respondents identified fiscal policy interventions (e.g., reduced taxes on healthy, sustainable foods such as vegetables and legumes), mandatory cooking and nutrition education in schools, and agricultural subsidies as most impactful (Figure 7). In contrast, distance norms between schools and fast-food outlets and role modeling by leaders (politicians, government officials, and managers) were rated as less influential.

**FIGURE 8:**
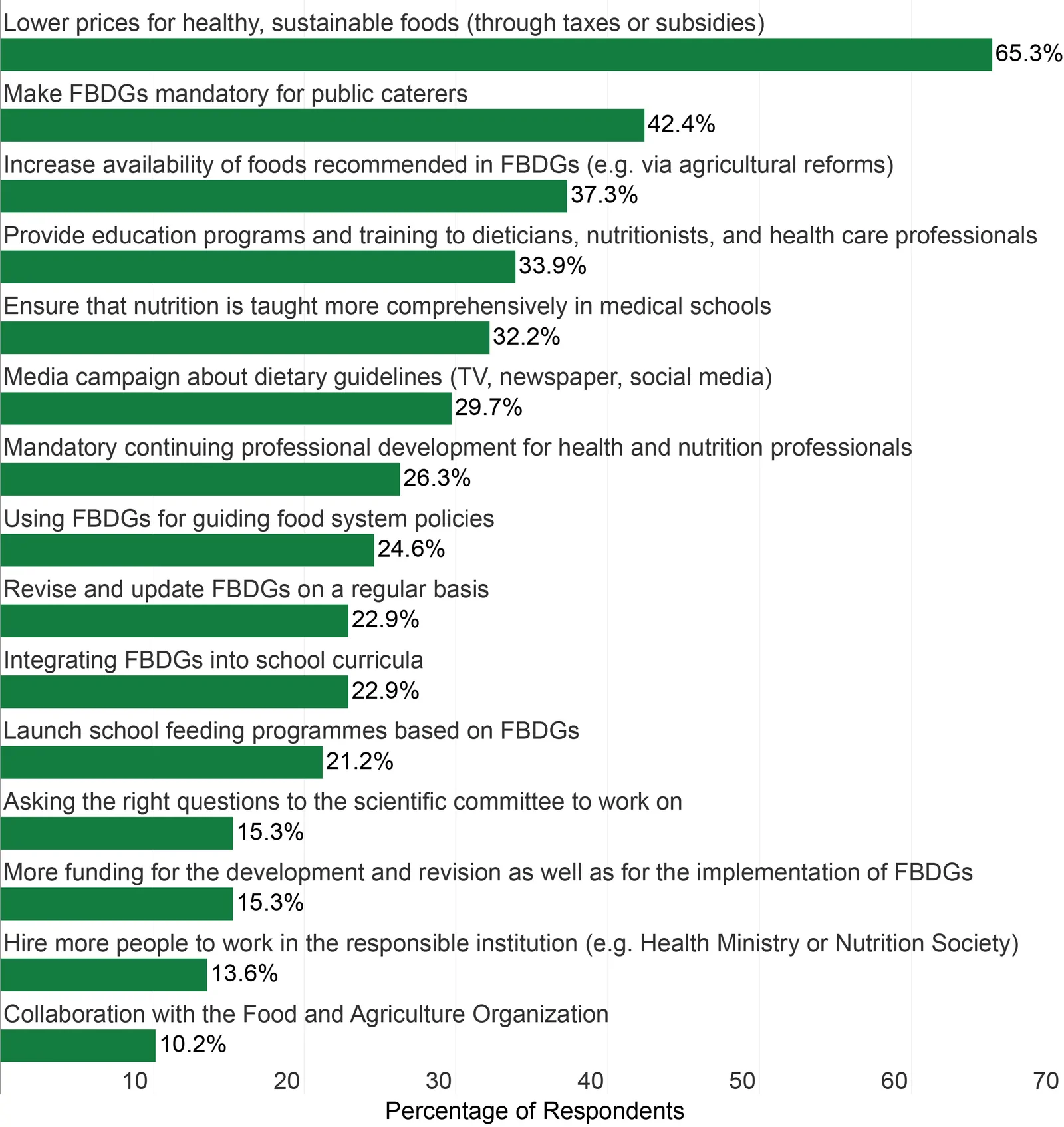
Priority actions for government to improve FBDG content and adherence. FBDG, food-based dietary guidelines.

When asked about the most effective governmental levers to improve FBDG content and adherence in the future, respondents highlighted lowering prices for healthy, sustainable foods through taxes or subsidies as the strongest measure (Figure 8). This was followed by making FBDG mandatory for public caterers (e.g., in schools, kindergartens, and hospitals) and increasing the availability of FBDG-recommended foods through agricultural reforms. Conversely, collaboration with the FAO or hiring additional staff within responsible institutions (e.g., Health Ministry or Nutrition Society) was considered less important.

## Discussion

This study is the first global analysis to explore the perspectives of nutrition and health professionals on the implementation of national FBDG. Using an SEF, we identified a complex interplay of intrapersonal, interpersonal, institutional, community, and policy-level factors that either facilitate or hinder the successful implementation of FBDG. The following sections discuss these findings across SEF levels, highlighting how barriers and enablers at higher systemic levels often constrain individual professional practice.

Our findings reveal a profound skepticism among health and nutrition professionals regarding the influence of the food industry on FBDG. A majority (64.4%) identified the power of the food industry to shape nutritional guidance as a “very influential barrier.” Within the SEF, these concerns reflect influences operating primarily at the public policy and community levels, where food systems, economic interests, and regulatory environments shape the content and credibility of dietary guidance. This perception might be rooted in documented cases where industry interests have shaped FBDG revision processes, softening or omitting advice on unhealthy or unsustainable foods [1,4,30,31]. Furthermore, a cross-country study examining correlations between the content of FBDG and a range of national characteristics found that countries in which meat production represents a larger share of gross domestic product tend to include fewer plant-based options in their dietary guidelines [4]. Conversely, stronger political commitment to environmental goals (measured using the Yale Environmental Performance Index) was associated with greater inclusion of plant-based options. These associations were statistically significant, indicating that broader economic and political contexts shape the framing of dietary guidance [4]. Although the FAO theoretically positions FBDG as guiding the private sector [32], existing literature suggests that this relationship is often reciprocal, with industry acting as a powerful counterinfluence [1,4,30,31].

This critical view is further linked to concerns about public misinformation. Our quantitative survey revealed that 47.5% of professionals considered common misconceptions about food and nutrition to be a “very influential barrier.” This is particularly relevant in the current digital age, where social media and uncredentialed “wellness experts” contribute to a widespread dissemination of misinformation [33]. At the community and interpersonal levels of the SEF, this directly affects professionals’ ability to translate FBDG into practice. Misinformation circulating through social networks makes the role of credible health and nutrition professionals, who are equipped to model critical thinking and information literacy, more vital than ever. As Diekman et al. [33] highlight, a practitioner’s ability to objectively analyze facts and apply information literacy skills is essential. To support this, we recommend that FBDG committees consider providing critical thinking frameworks and checklists for ethical practice, similar to those proposed by Diekman and colleagues, to help professionals navigate this complex information landscape.

Despite these challenges, our qualitative interviews also highlighted positive examples of industry-led initiatives, such as healthy checkout aisles in South Africa and FBDG-aligned school catering in Germany. These cases illustrate the potential for industry to become a powerful ally in FBDG implementation. Given that populations globally rely heavily on commercial food systems, the industry has a responsibility to align its business strategies with public health goals [34]. By developing and marketing products consistent with FBDG, providing transparent labeling, and making healthier options more accessible, the private sector can actively support the shift toward healthy, sustainable diets [[35], [36], [37]]. The dual role of the food industry—as both a barrier and a potential enabler—illustrates how community- and institutional-level actors can either undermine or support individual-level implementation efforts.

Our study found that professionals strongly advocate for systemic change, particularly through government policy. This aligns with the finding that the socioeconomic status of patients and food costs were substantial barriers. Participants expressed a strong desire for FBDG to include recommendations for achieving a healthy diet on a budget, and they see politicians as having a responsibility to use fiscal and regulatory policies to support this shift. For instance, interventions like agricultural subsidies that favor healthy, sustainable foods and reduced taxes on items like vegetables and legumes were identified as essential for effective FBDG implementation. This perspective is supported by existing evidence showing that food prices significantly influence dietary choices, especially for low-income individuals [34,37]. The professionals surveyed do not place the burden of healthy eating solely on the individual but instead call for a supportive food environment enabled by government action. This framing aligns with the SEF assumption that behavior change is unlikely without supportive structural and policy conditions.

A central finding of this study is that professionals perceive the FBDG themselves as only “somewhat” or “slightly helpful” in promoting healthy, sustainable food choices (57.62% of respondents). One notable subgroup finding was that professionals from LMICs perceived their national FBDG as more helpful than professionals from HICs. Although the reasons for this difference cannot be determined from the present study, it may suggest that FBDG play a comparatively greater role as a practical nutrition resource in settings where fewer alternative evidence-based nutrition resources or clinical guidance documents are readily available. Alternatively, it may reflect differences in the content, implementation, or perceived relevance of national FBDG across countries.

Within the SEF, the design and content of FBDG operate at the policy level while directly influencing intrapersonal factors such as professionals’ confidence, perceived relevance, and willingness to implement recommendations. Our findings suggest that the intrinsic characteristics of the guidelines are a barrier to their implementation. A key concern is that many FBDG are outdated and do not reflect the latest scientific evidence, with professionals feeling uncomfortable implementing recommendations that are not current. This might be particularly true for guidelines that underrepresent plant-based foods while giving disproportionate emphasis to animal-based products like meat, dairy, and eggs, as identified by our survey and confirmed by a broader analysis of FBDG [1,3,4,38].

Legumes (62.7%), plant-based milk alternatives (47.5%), and nuts and seeds (42.4%) were most frequently identified as underrepresented in the current FBDG. Given the growing public interest in plant-based alternatives and the expanding evidence base on their health and environmental implications [[39], [40], [41]], a large majority of participants supported the explicit inclusion of these products in dietary guidelines. Together, these findings underscore the need for FBDG to remain responsive to evolving dietary patterns and broader cultural shifts [38], and highlight a misalignment between existing institutional guidance and contemporary community-level food practices.

Although foods such as tofu, soya milk, and seitan have long been integral to certain traditional diets, their consumption is increasing globally, alongside a rapidly expanding market for novel plant-based alternatives [[39], [40], [41]]. Rising health awareness and consumer acceptance have driven substantial growth in sales and investment, particularly in Europe, North America, and Asia [39,41]. These trends highlight the need for FBDG to move beyond static food categorizations and provide guidance that reflects contemporary food environments.

The German Nutrition Society’s (DGE) position paper on plant-based milk alternatives illustrates how FBDG can remain culturally relevant while responding to changing consumption patterns [42,43]. Recognizing the increasing use of these products, the DGE recommends prioritizing fortified plant-based alternatives with key nutrients—particularly calcium, iodine, and vitamins B2 and B12—or ensuring adequate intake from alternative sources such as green leafy vegetables, iodized salt, or supplements [42,43]. Explicit guidance within FBDG could therefore not only support consumers and health professionals in preventing potential nutrient inadequacies but also incentivize manufacturers to improve the nutritional quality of plant-based milk alternatives [38].

More broadly, several countries—including Belgium [44], Germany [45], Mexico [46], and the Nordic countries [47]—have revised their FBDG in recent years to better promote healthy and sustainable diets by aligning with the Planetary Health Diet proposed by the EAT-Lancet Commission [48]. This global framework emphasizes a plant-forward diet with animal-source foods occupying a considerably smaller proportion, recognizing the interconnectedness of human and planetary health [48,49].

Furthermore, the design and format of FBDG matter for their usability. Although there are over 70 different graphical formats [50], our participants overwhelmingly preferred the plate model over the pyramid, citing its ease of understandability. This preference is consistent with studies from Australia and Canada, where updated guidelines using a plate model were perceived as clearer and aesthetically pleasing by the public [51,52]. However, the lack of sufficient, up-to-date data on how different visuals are understood by various age groups and cultural contexts highlights a need for further research [50,53].

Although FBDG are typically designed to be simple and accessible for the general public [[54], [55], [56]], our findings suggest that this level of detail often does not meet the needs of health professionals responsible for implementing the guidelines. A dual-format strategy—a simplified public version and a more detailed professional resource—was strongly supported by participants (84.75%) and is already used in countries like Australia [57] and Sri Lanka [58]. Such an approach could improve implementation while maintaining public accessibility.

Until the update in 2026, the United States also provided additional supporting materials specifically designed for healthcare professionals; however, these are no longer included in the current version of the Dietary Guidelines for Americans (DGA). Compared with previous editions, the new publication contains fewer “ready-to-use” resources for patient counseling, such as tip sheets [59].

Culture emerged as a substantial factor influencing FBDG implementation. At the interpersonal and community levels of the SEF, cultural norms, traditions, and social identities shape both food choices and professional–client interactions. Although FBDG around the world share remarkable similarities despite vast cultural differences [2,60,61], our findings highlight that a successful implementation strategy must be culturally sensitive and inclusive. We found that health and nutrition professionals frequently encounter cultural influences that act as both assets and barriers to healthy eating.

Our findings support a move toward more culturally tailored FBDG. The 2025 United States Dietary Guidelines Advisory Committee, for example, used a “health equity lens” to consider factors such as socioeconomic position, race, and ethnicity [62]. They were the first committee to employ diet simulations, a systems science approach, to evaluate proposed dietary patterns while accounting for variability in food choices across different cultures and traditions [62]. However, it should be noted that the final 2025–2030 DGA, released in January 2026 under the Trump administration, did not incorporate this specific scientific advice from the Advisory Committee [59].

Another crucial aspect of cultural sensitivity is the acknowledgment and integration of indigenous foods [63,64]. Countries like Brazil and Canada have incorporated these traditional foods into their national guidelines, recognizing their role in developing sustainable and equitable food systems [63,64].

In the context of cultural influences, it is also important to consider varying nutritional philosophies [4]. This applies to both citizens and professionals themselves. Our survey revealed that a majority of health and nutrition professionals follow some form of plant-based diet (especially whole food plant-based diet, Mediterranean diet, vegan diet, and planetary health diet), reflecting a growing global interest driven by concerns for health, environmental sustainability, and animal welfare. This finding illustrates how intrapersonal values and beliefs among professionals can interact with institutional guidance, either reinforcing or challenging existing FBDG frameworks. With ∼1.5 billion people globally adhering to some form of vegetarian dietary pattern [6], a considerable information gap exists, as 60% of FBDG worldwide do not currently provide guidance for these diets [4]. The overwhelming majority of our respondents (90.8%) agreed that FBDG should provide information for well-planned vegetarian and vegan diets. This suggests that guidelines with an inclusive design that account for diverse nutritional philosophies can increase their relevance and, in turn, the likelihood of their implementation by professionals.

Successful FBDG implementation is also contingent on a well-trained workforce and transparent governance. Our findings show that although dietitians are generally well-versed in FBDG, physicians report receiving little to no formal nutrition education during their medical training. This is a critical gap, given that doctors are often the first point of contact for patients with nutrition-related concerns. Insufficient nutrition training among medical professionals is a widely documented global issue and remains a substantial barrier to effective dietary counseling.

Our results are consistent with those of Crowley et al. [65], who reported low nutrition knowledge and confidence among medical students worldwide despite strong interest in the subject, pointing to a persistent disconnect between educational demand and curricular provision. Addressing this gap requires coordinated action, including curriculum reform and policy-level engagement. Collaboration between Ministries of Health, accreditation bodies, and medical schools is essential to ensure that nutrition education is embedded within core medical training and linked to accreditation standards.

Beyond individual knowledge gaps, our findings corroborate evidence that institutional support plays a decisive role in enabling health professionals to integrate sustainable nutrition into practice. This reinforces the SEF assumption that individual competence alone is insufficient without supportive institutional and policy environments. In line with Guillaumie et al. [18], participants emphasized that perceived knowledge must be complemented by clear and actionable guidelines, practical implementation tools, and enabling institutional conditions. This is further supported by Muñoz-Martínez et al. [17], who found that although many dietitians recognize the importance of sustainability, relatively few feel confident in defining or applying sustainable dietary principles in their professional practice. These shortcomings have been attributed to limited training opportunities and insufficient structural support, including the absence of up-to-date, sustainability-oriented FBDG. Our findings confirm and extend this literature by elucidating the specific role of FBDG as both a knowledge resource and an enabling instrument shaping professional practice, while also highlighting opportunities to strengthen implementation through targeted support and policy measures.

Governance of FBDG emerged as an equally critical factor. Nearly all participants (99.1%) rated transparency in the development and revision of FBDG as “particularly important,” underscoring the central role of good governance practices. Good governance practices, which include transparency, participation, and inclusivity, are strongly linked to increased public acceptance of policies [[66], [67], [68], [69]]. It is plausible that professionals are more willing to implement FBDG if they feel that the process is fair and their input is valued. From a socioecological perspective, transparent and participatory governance represents a policy-level mechanism that strengthens institutional trust and facilitates individual professional engagement with FBDG.

Public consultations can serve as a crucial mechanism for this. They can strengthen the development and implementation of FBDG by increasing transparency, incorporating diverse stakeholder perspectives, and building trust in the final recommendations. Countries such as Canada [70], Australia [71], the Nordic countries [72], and the United States [73] have already implemented such processes, but the way they are organized varies. The Canadian food guide, for example, successfully applied a framework of “good governance of evidence,” integrating both scientific and democratic principles [74]. Such transparent and participatory approaches can enhance the perceived legitimacy and relevance of FBDG among health professionals, thereby supporting their translation from policy into practice.

### Strengths and limitations

This study’s exploratory sequential mixed-methods design is a significant strength. By using qualitative interviews to inform the quantitative survey, the study ensured that the survey questions were grounded in the real-world experiences and perspectives of health and nutrition professionals. This approach enhances the validity and relevance of the quantitative findings, as the survey items directly reflect the barriers and facilitators identified by the participants in the initial phase. The use of the SEF as a coding structure provides a robust theoretical foundation, allowing for a systematic analysis of the multifaceted factors influencing FBDG implementation across various levels.

The inclusion of health and nutrition professionals from six continents represents a further important strength. Although the sample is not statistically representative, this broad geographical coverage offers a rare global perspective on FBDG implementation, capturing commonalities and divergences across diverse policy, cultural, and food system contexts. Given the global relevance of FBDG and the increasing convergence of dietary guidance around health and sustainability goals, this international scope strengthens the interpretive value of the findings and supports their relevance beyond single-country analyses.

Despite these strengths, several limitations must be considered. The study’s nonrepresentative sample is a primary constraint. Because of the use of convenience and snowball sampling, the participants may not be fully representative of the broader population of health and nutrition professionals, which limits the generalizability of the findings. The sample was small (*n* = 20 for qualitative, *n* = 118 for quantitative), particularly for a global study, which may not capture the full diversity of views and experiences. The overrepresentation of dietitians and medical doctors in the qualitative phase may also introduce a bias toward their specific professional perspectives, potentially underrepresenting other health and nutrition roles. The self-reported nature of the survey data is another limitation, because it is susceptible to social desirability bias, where participants may provide responses they believe are expected rather than their true views or behaviors. Finally, the recruitment method may have disproportionately attracted professionals with a high level of interest and engagement in FBDG, potentially leading to findings that are not reflective of the attitudes of less-engaged professionals.

## Conclusion

Nutrition and health professionals face multiple barriers and facilitators when implementing national FBDG, spanning intrapersonal, interpersonal, institutional, community, and policy levels. Improving implementation, therefore, requires more than evidence-based dietary recommendations alone; it demands coordinated action across the wider food system. Policymakers, government-appointed scientific committees, educational institutions, healthcare organizations, food manufacturers, retailers, and other food-system stakeholders all have a role in strengthening nutrition education, ensuring transparent and participatory guideline development, providing practical implementation resources, and creating food environments that make healthy and sustainable choices easier. By addressing these factors collectively, FBDG can evolve from evidence-based policy documents into effective instruments for improving population health and accelerating the transition toward healthier and more sustainable food systems. Future research should build on these findings by evaluating implementation strategies across different national and cultural contexts.

## Author contributions

The authors’ responsibilities were as follows – A-LK: designed the research (project conception, development of overall research plan, and study oversight), conducted the research (data collection), wrote the paper, and primarily responsible for final content; A-LK, JPS, VG, DABB: analyzed the data and performed statistical analysis; AR: provided guidance on the study frame, conduction, and reviewed the manuscript; and all authors: read and approved the final manuscript.

## Data availability

Data described in the manuscript, code book, and analytic code will be made available upon request to the corresponding author.

## Declaration of generative AI and AI-assisted technologies in the writing process

During the preparation of this work, the authors used Gemini and DeepL to edit the manuscript for clarity and language, as well as to assist in writing code for data analysis with Python. After using these tools/services, the authors reviewed and edited the content as needed and take full responsibility for the content of the publication.

## Funding

This research was financially supported by the Federal Ministry of Research, Space and Technology Germany (BMFTR), ProVeg International, and the Open Access Publication Funds of the Göttingen University. The funding sources had no role in the study design; in the collection, analysis, and interpretation of data; in the writing of the manuscript; or in the decision to submit the article for publication.

## Conflict of interest

A-LK, VG, and DABB are employees with ProVeg International, a nongovernmental organization; A-LK is also a PhD candidate at the University of Göttingen. AR and JPS declare no competing interests. All opinions presented in this article belong to the authors alone and not to any organization with which they are or were affiliated.
