# Supplementary material for: From Policy to Practice: A Global Analysis to Identify Factors That Influence the Implementation of Food-Based Dietary Guidelines by Health and Nutrition Professionals
Source: Curr Dev Nutr. 2026 Aug 7;10(9):109509. doi: 10.1016/j.cdnut.2026.109509 (PMC13564136; doi:10.1016/j.cdnut.2026.109509)
Supplement: Supplementary file 1 [file mmc1.pdf]

Supplementary File 1: Guide for Qualitative Interviews

Klapp et al. (2026): From policy to practice: A global analysis to identify factors that influence the implementation of food-based dietary guidelines by health and nutrition professionals

**Interview phase/question type**	**Text**
Welcome	Hello, my name is XY.<br><br>You may know that there has been growing interest in food-based dietary guidelines (FBDG) by several researchers from different disciplines since these guidelines are recognized as important tools for establishing healthier and more sustainable diets. In this context, FAO, WHO, and IPCC advocate the development and revision of national dietary guidelines to provide context-specific recommendations for sustainable healthy diets.<br><br>Today, I would like to talk to you about your expectations regarding dietary guidelines and the responsible institutions.<br><br>The interview's main aim is to determine the expectations and whether the guidelines and the responsible institutions meet these expectations.<br><br>What factors influence the implementation of dietary guidelines, and what is needed for nutrition and health professionals to be able to support healthy, sustainable diets?
Consent request	Do you agree with the interview being recorded?
Initial question	Please tell me why and in which context you have dealt with dietary guidelines in the past.

Key questions	When you think about the design of an FBDG and the information it provides, what are your expectations of a guideline that aims to promote healthy, sustainable food choices?<br><br>In your opinion, which expectations regarding FBDGs are currently not fulfilled at all?<br><br>What factors influence the implementation of dietary guidelines by health and nutrition professionals?<br><br>Can you please describe what the training provided by the responsible institution for the FBDG looks like?<br><br>Does this training meet your expectations/what do these expectations look like?<br><br>What is particularly positive or negative about the government's approach?<br><br>Do you trust the responsible authority for FBDGs to provide the necessary resources and information to promote healthy, sustainable food choices? Why?<br><br>What else is needed to promote healthy, sustainable food choices?<br><br>What should governments do in the future to improve FBDGs and training with FBDGs?
Statements that can help revive the conversation	Feel free to think about this for a while…<br>Why?<br>What do you think about that?<br>What else can you tell me about it?
Revisit the past	So, you mentioned earlier that…<br>Earlier, you said that…<br>Getting back to…
Additional question	Which other person should I interview?

Goodbye	Thank you for taking the time to answer my questions. If you have any further questions, please do not hesitate to contact me! Goodbye!
