# Supplementary material for: From Policy to Practice: A Global Analysis to Identify Factors That Influence the Implementation of Food-Based Dietary Guidelines by Health and Nutrition Professionals
Source: Curr Dev Nutr. 2026 Aug 7;10(9):109509. doi: 10.1016/j.cdnut.2026.109509 (PMC13564136; doi:10.1016/j.cdnut.2026.109509)
Supplement: Supplementary file 2 [file mmc2.pdf]

Supplementary File 2: Set of questions for a quantitative online survey

Klapp et al. (2026): From policy to practice: A global analysis to identify factors that influence the implementation of food-based dietary guidelines by health and nutrition professionals

---

Professional background

This survey aims to better understand the **expectations of nutrition and health** professionals concerning food-based dietary guidelines. To get started, we would like to know **your** professional background. Therefore, it is important that we know **what discipline you come from** and **what area you work in**.

1. Please select your current primary role: (select one)

o Physician / medical doctor
o Nurse
o Medical Assistant
o Dietician
o Nutritionist
o Epidemiologist
o Researcher
o Program Director / Staff
o Policy / Advocate
o Government Official
o Consultant Public Affairs
o Author / Journalist
o Other (please describe) ______________________________

2. What is your educational background? (select one)

o Health Sciences
o Medicine
o Nutritional Sciences
o Dietetics
o Public Health
o Public Health Nutrition
o Epidemiology
o Pharmacy
o Agricultural Sciences
o Food Technology
o Psychology
o Consumer Science
o Other (please describe) ______________________________

3. What best describes the type of organization you are working for? (select one)

- o Academia
- o Education
- o Non-Governmental Organization (e.g. non-profit, foundation)
- o Advocacy Organization
- o Government
- o Hospital, clinic, or medical center
- o Nursing home
- o Pharmacy
- o Law
- o Private Industry
- o Media
- o Self-employed
- o Health insurance company
- o National Nutrition Society
- o Other (please describe) ______________________________

4. Where do you live currently? (select one country)

⇒ *Drop-down menu with countries*

5. There are various dietary concepts and nutrition philosophies that nutrition and health experts might be connected to. These are dietary patterns that can be followed by individuals, or used, for example, to guide their nutritional counselling. We would like to know which of the following dietary concepts or nutrition philosophies you feel most connected to. Please select one.

- o Whole food plant-based diet
- o Ayurvedic Diet
- o Mediterranean diet
- o Intermittent fasting
- o Low-carbohydrate diet
- o Low-fat diet
- o Vegan diet
- o Vegetarian diet
- o Planetary Health Diet
- o Religious diet (e.g. Buddhist, Christian, Hindu, Islamic, Jain, Jewish, etc.)
- o Chinese food therapy
- o DASH diet (Dietary Approaches to Stop Hypertension)
- o Clean eating
- o Paleo diet
- o Raw food diet
- o I don’t feel connected to a specific dietary concept or nutrition philosophy

o Other (please describe) ____

Food-based dietary guidelines (FBDGs)

Some may remember the dietary guidelines as a **food pyramid that advises citizens about healthy eating**. However, national food-based dietary guidelines (FBDGs) are more than that. In addition to providing advice about healthy eating, these guidelines form the basis for the development of official food policies as well as those related to nutrition and health programs. They serve as an **information source for health and nutrition professionals**, and form the basis for catering recommendations in institutions such as schools, rehabilitation clinics, and hospitals, as well as inpatient facilities for the elderly.

Worldwide, more than 100 countries leverage FBDGs, and the Food and Agricultural Organization of the UN (FAO) underscores the **important socio-political relevance** of dietary guidelines.

Please tell us, were food-based dietary guidelines (FBDGs) part of your studies (university, medical school, etc.)?

o Yes, they were discussed in detail
o Yes, but they were only mentioned briefly
o No
o I can not remember

Do you use the national FBDG of the country you are based in for your work? For example, in nutritional counseling with your clients or in public lectures.

o Yes
o Sometimes
o No

(Follow-up question)
If you don't or just sometimes use the national FBDG of the country you are based in, which guideline do you use instead?

o From another country
o EAT-Lancet Planetary Health Diet
o American College of Lifestyle Medicine
o A self-created one
o World Health Organization (WHO) guidelines
o Guidelines of the institution I work for
o Healthy Eating Plate created by Harvard School of Public Health
o None
o Other (please describe) ____

(Follow-up question)
If you use the FBDG of another country, which country is it?
⇒ *Add drop-down menu with countries here*

Expectations

Food-based dietary guidelines (FBDG) are recognized as **important tools for establishing healthier and more sustainable diets**. In this context, FAO, WHO, and IPCC advocate the development and revision of national dietary guidelines to **provide context-specific recommendations** for sustainable healthy diets.

What are your expectations of an FBDG that promotes healthy and sustainable food choices? Please select the 3 most important aspects for you.

- o It should be based on the latest scientific evidence
- o It should give recommendations on what will either improve, manage, or prevent diseases
- o It should include food that is available to the majority of the citizens for which it is written for
- o It should reflect the barriers people face when choosing healthy, sustainable foods
- o It should be applicable to clinical practice
- o It should include myth-busting (prove/explain that something that is generally thought to be true is not)
- o It should tell people what is optimal for health
- o It should inform people about which food groups have the least impact on the environment
- o It should break information down with infographics
- o It should include recommendations on how to plan a vegetarian and vegan diet well
- o It should be flexible and inclusive
- o An adaptation to the local context should be possible
- o It should include recommendations on the food system level (e.g. what the Ministry of Agriculture can do to provide more fruits and vegetables)
- o It should include a clear message to reduce the consumption of meat
- o It should include a clear message to eat more plant-based foods

Which expectations regarding FBDGs are currently <u>not fulfilled at all</u>? Please select the 3 most important aspects for you.
- o Situational awareness (the ability to perceive, understand, and effectively respond to the situation of citizens)
- o Inclusion of ecological sustainability
- o Concrete statements instead of vague information
- o Inclusion of behavioral change strategies

- o Taking current eating patterns of the population into account
- o Easy-to-understand food graphic (e.g. plate, pyramide, basket, etc.)
- o Practical recommendations such as recipes
- o Information on healthy, sustainable diets on a budget
- o Recommendations adapted to different cultures
- o Recommendations on the food system level (e.g. what the Ministry of Agriculture can do to provide more fruits and vegetables)

On a scale of 1 to 5, how helpful do you consider the national FBDG
<u>in the country you are located in</u> to promote healthy sustainable food choices?
Note that 1 is not at all helpful and 5 is extremely helpful.

You can find an overview of national dietary guidelines from more than 100 countries here: https://www.fao.org/nutrition/education/food-based-dietary-guidelines

Please be aware that the FAO website may not show the latest guidelines for your country. In this case, you should go to the website of the responsible authority in your country. This link is also provided on the FAO website.

**1**	**2**	**3**	**4**	**5**	**6**
Extremely helpful	Very helpful	Somewhat helpful	Slightly helpful	Not at all helpful	The country I am located in has no dietary guideline

There are basically two different approaches to modelling an FBDG:
(1) Optimal: recommendations that show what an optimal diet should look like.
(2) Feasible: recommendations that are closer to the current dietary patterns of the population which means they include also foods that are considered as less healthy.

Which approach do you find most helpfull for your work (for example, in the counselling situation with your patient)?

- o Approach 1: Optimal diet
- o Approach 2: Feasible diet
- o I don't use the guidelines for my work
- o I don't know

Food graphics

The **main messages** of the FBDG are often presented via **visual representations such as plates, pyramids, or other diagrams**. These food graphics are supposed to provide an **official and simple way** to address the idea of healthy eating and often function as a representation of the entire guideline.

We want to know, what is your favorite shape?

- o Pyramid
- o Plate / Circle
- o Building/Structure (house, pagoda, tent)
- o Fruit (pineapple, breadfruit)
- o Basket
- o Other: _________

Why is this shape your favorite one?

- o Easy to understand
- o Closer to the reality of people's lives
- o It makes clear which foods should be eaten frequently and which less often.
- o Other: _______

In your opinion, which food is the most underrepresented food so far? Choose up to three foods, you can also choose just one or two.

- o Meat
- o Eggs
- o Milk and dairy products
- o Fish
- o Pulses, beans, and legumes
- o Whole grain and whole grain products
- o Refined grains and refined grain products
- o Plant-based milk alternatives
- o Plant-based meat alternatives
- o Fruits
- o Vegetables
- o Starchy roots (e.g. potato, yam, sunchoke, etc.)
- o Nuts and seeds
- o Herbs

o Oil

In your opinion, which food is the most <u>over-represented</u> food so far? Choose up to three foods, you can also choose just one or two.

o Meat
o Eggs
o Milk and dairy products
o Fish
o Pulses, beans, and legumes
o Whole grain and whole grain products
o Refined grains and refined grain products
o Plant-based milk alternatives
o Plant-based meat alternatives
o Fruits
o Vegetables
o Starchy roots (e.g. potato, yam, sunchoke, etc.)
o Nuts and seeds
o Herbs
o Oil

Content and structure of FBDGs

Dietary guidelines **categorise foods into food groups**. There are different approaches from country to country. Each food group should be part of one's diet, but different foods can be chosen within a group. Therefore **it is key how foods are grouped together.**

On a scale of 1 to 5, how helpful do you consider the grouping of foods in the following examples from existing guidelines for promoting sustainable healthy food choices? Note that 1 is not at all helpful and 5 is extremely helpful.

Example with 7 Food groups:
Cereals, cereal products, potatoes
Vegetables and salad
Fruits
Milk and dairy products
Meat, sausage, fish and eggs
Oils and fats
Drinks

1	2	3	4	5
Extremely helpful	Very helpful	Moderately helpful	Slightly helpful	Not at all helpful

Example with 6 Food groups:
Vegetables
Fruit
Cereals & Starchy Vegetables
Legumes
Milk, Dairy Products & Plant-Based Alternatives
Fish, Poultry, Meat & Plant-Based Alternatives

1	2	3	4	5
Extremely helpful	Very helpful	Moderately helpful	Slightly helpful	Not at all helpful

Example with 5 Food groups:
Vegetables and Fruits
Bread, grain/cereal products and potatoes
Dairy, nuts, fish, legumes, meat and eggs
Spreading and cooking fats
Drinks

1	2	3	4	5
Extremely helpful	Very helpful	Moderately helpful	Slightly helpful	Not at all helpful

Example with 4 Food groups:
Vegetables and Fruits
Protein
Whole Grain
Water

1	2	3	4	5
Extremely helpful	Very helpful	Moderately helpful	Slightly helpful	Not at all helpful

The following statements were taken from actual existing national dietary guidelines and paraphrased. Please rate each statement based on how helpful you think it is in promoting healthy sustainable food choices.

	1	2	3	4	5
**Statement**	Very helpful	helpful	neither nor	not helpful	Not at all helpful
Eat either fish, poultry, meat, or eggs every day.					
Eat animal-based milk and dairy products every day.					
Meat, maximum of 3 servings per week.					
Reduced consumption and thus production of animal-based foods will reduce emissions of greenhouse gases, of deforestation caused by creation of new grazing areas for cattle, and of intensive use of water.					
Eat legumes daily. Legumes are a good source of plant-based protein and, in combination with cereals in our daily diet, provide essential amino acids to our body.					
Choose whole grain foods instead of refined grains.					
Choose protein foods that come from plants more often.					
Eat plenty of vegetables and fruit every day.					
Drinks made of oats and soya are eco-friendly. Choose the ones enriched with vitamins and minerals – you'll see this information on the packaging.					

Fortified soy milk and soy yogurt are included in the dairy group because their nutrient composition and use in meals make them comparable to milk and yogurt.					
It is important to know that consuming a cup of soy juice, also misnamed as ‘soy milk’, is not the same as drinking a cup of cow’s milk because soy juice does not contain the necessary amounts of nutrients, mainly the amount of calcium, to cover the needs of the organism. Therefore, it is always better to consume cow’s milk if it is available.					
Eat less meat and more plant-based foods. Vary with fish, pulses, nuts, and eggs. Plant-based meat substitutes can also be a good source of protein. Choose substitutes with low or moderate salt and fat content.					
A healthy and sustainable diet should reduce and even avoid processed foods high in sugars, fats and salt.					
One cannot be a healthy vegetarian by going to a fast food restaurant and ordering french fries and soda! Vegetarians can meet their nutrient needs by choosing a variety of meat alternatives such as beans, lentils, eggs, tofu, soy-based meat substitutes, nuts, nut butters and seeds.					
A plant-based diet is associated with a lower risk of obesity, cardiovascular disease, diabetes, and cancer. With good knowledge and planning, both vegetarian and vegan diets can be suitable for people in all phases of life, including during pregnancy and breastfeeding, for infants, for children, and adolescents and for athletes.					
Vegan and vegetarian diets have, among other things, significance for the climate impact of diet. Vegan diets generally provide the greatest reduction in climate footprint, followed by vegetarian diets and plant-rich diets with less meat and dairy products.					

Vegan diets are extremely restricted due to the absence of all animal-based products (i.e. also eggs, milk and honey), which can lead to a risk of insufficient intake of various nutrients and protective substances. However, with a specific and careful choice of food, it is possible to achieve an intake that meets requirements. In certain phases of life (e.g. pregnancy, breastfeeding, childhood, growth, old age), however, complete coverage is more difficult. Therefore, the vegan diet is not suitable for the general population.					

Macronutrients and Micronutrients

FBDGs provide recommendations on **foods as sources of macronutrients** (carbohydrates, proteins, fats) and **micronutrients** (vitamins and minerals).

However, all large-scale evaluations of FBDGs have shown that governments are **lagging behind in integrating health and sustainability goals** into national dietary guidelines and often only recommend resource-intensive animal-based foods for certain nutrients.

Keeping in mind the support for sustainable, healthy food choices, please tell us your opinion on the following six statements.

FBDGs should include recommendations on plant-based protein sources such as legumes, beans, nuts, seeds, tofu, and soya milk. They should also explain the importance of combining different plant-based foods to achieve a complete profile of amino acids over the day, for example, rice and beans.

1	2	3	4	5
Strongly disagree	Disagree	Undecided	Agree	Strongly agree

Meat is often mentioned as the only source of iron. FBDGs should also provide information on how to meet iron requirements from plant-based sources, for example, by consuming soaked and cooked legumes with vitamin-c-rich food which promotes bioavailability efficiency.

1	2	3	4	5
Strongly disagree	Disagree	Undecided	Agree	Strongly agree

Cow's milk and dairy products are often mentioned as the only source of calcium to improve bone mineral density. FBDGs should also inform people that they can meet their calcium needs with plant-based foods that have a high calcium bioavailability, such as certain green leafy vegetables (e.g. kale, broccoli, and turnip greens), calcium-rich mineral water, or unsweetened calcium-fortified plant-based milk.

1	2	3	4	5
Strongly disagree	Disagree	Undecided	Agree	Strongly agree

Fish is often mentioned as the only source of omega-3 fatty acids. While some fish (mainly cold-water fish) are the main source of the long-chain omega-3s EPA and DHA, FBDGs should explain, that the main sources of the short-chain omega-3 ALA are plant oils (canola, flax, and soybean), nuts (especially walnuts), and seeds (especially chia and flax seeds).
In this context, FBDGs should provide additional information on how to meet EPA and DHA requirements with plant-based sources, for example by doubling the ALA intake.

1	2	3	4	5
Strongly disagree	Disagree	Undecided	Agree	Strongly agree

FBDGs should include explicit recommendations for adequate vitamin B12 supply for people who consume few or no animal-based products. For example, to take dietary supplements in order to ensure an adequate supply of vitamin B12.

1	2	3	4	5
Strongly disagree	Disagree	Undecided	Agree	Strongly agree

Plant-based Alternatives

Public **interest in plant-based alternatives** has increased rapidly in the last few years along with the number of scientific articles about their benefits and limitations. Please tell us **your opinion** on the following three statements.

Plant-based meat alternatives such as tofu, seitan, and veggie meat should be included in FBDGs. This should be combined with information such as "prefer products low in salt and fat".

1	2	3	4	5
Strongly disagree	Disagree	Undecided	Agree	Strongly agree

Plant-based milk alternatives such as soya milk, oat milk, or almond milk should be included in FBDGs. This should be combined with information such as "prefer products without added sugar" and “prefer calcium-enriched plant-based milk”.

1	2	3	4	5
Strongly disagree	Disagree	Undecided	Agree	Strongly agree

Plant-based dairy alternatives such as soya yogurt and nut cheese should be included in FBDGs. This should be combined with information such as "prefer products low in saturated fat".

1	2	3	4	5
Strongly disagree	Disagree	Undecided	Agree	Strongly agree

Inclusion and diversity

The main target group of an FBDG is the general population. However, it can also go **beyond this target group** and try to **be inclusive and diverse**. We would like to hear your opinion on the following two statements.

FBDGs should balance the ethical, ecological, religious, and economic aspects that play a role in people's food choices.

1	2	3	4	5
Strongly disagree	Disagree	Undecided	Agree	Strongly agree

Besides recommendations for the general population, FBDGs should provide information for well-planned vegetarian and vegan diets.

1	2	3	4	5
Strongly disagree	Disagree	Undecided	Agree	Strongly agree

Implementation

Nutrition and health professionals are usually required to implement the FBDG of their country in their daily work. This can be made easier or more difficult by various aspects.

How helpful would you find it if there was an easy-to-understand FBDG for the general public and a second detailed FBDG for professionals? Note that 1 is not at all helpful and 5 is extremely helpful.

1	2	3	4	5
Extremely helpful	Very helpful	Somewhat helpful	Slightly helpful	Not at all helpful

There are several barriers to the implementation of FBDGs by nutrition and health professionals. Please select the appropriate option for each statement.

**Statement**	**Influences implementation not at all**	**rather influences implementation**	**neutral**	**influences implementation rather strongly**	**influences implementation very strongly**	**I can't assess this**
Lack of knowledge on the side of the health and nutrition professionals						
Resistance on the side of nutrition and health experts						
Understaffing in healthcare institutions						
Shortage of skilled workers (e.g. dieticians)						
Lack of time (as to how long you have to spend with the patient)						
The budget allocation						
Patient engagement						
Lack of literacy among general population						

No funding for an FBDG implementation campaign by the government						
Cultural influences on food habits (e.g. meat as a symbol of wealth and masculinity)						
Lack of availability and affordability of healthy foods						
Socio-economic status of the patient						
The power of the food industry to influence nutritional guidance						
Common misconceptions about food and nutrition						
No supporting material about the FBDG (e.g. flyers that can be given to patients)						
Insufficient or no collaboration between health institutions (e.g. hospitals) and the authority responsible for the FBDG						

Government and responsible authority

FBDGs are **government-written or government-commissioned documents.** In some countries the Ministry of Health is responsible, in other countries the government appoints a nutrition society which is responsible for the FBDG. We would like to know your opinion on these institutions and their activities.

Do you know if the responsible authority (for example the Ministry of Health or the Nutrition Society) or the government offers training programmes with the FBDGs for health and nutrition professionals?

o Yes, they do.

o No, I have never heard of this before

o I am not sure

Would you take part in such training if it was officially offered to nutrition and health professionals in the country where you are based?

o Yes, basically
o No, I have no interest in this
o I am not sure

How important do you consider the following aspects to ensure good governance with regard to FBDGs?

**Statement**	**Extremely important**	**Very important**	**Somewhat important**	**Slightly important**	**Not at all important**	**I can't assess this**
The members of the scientific committee have to declare a conflict of interest statement						
The FBDG development/revision process must be transparent						
The decision-makers are informed on the latest scientific evidence						
Public participation in the committee's review process is encouraged (e.g., offering public consultations for citizens, experts, and NGOs)						
No industry involvement						
Preventing and combating corruption						
Accountability of officials, public servants and scientists involved in						

the revision or development of the FBDG						
Activities regarding the FBDG should be predictable, efficient and effective.						
Protection of interests and rights of citizens by giving inclusive recommendations						
Evaluation and impact assessment of the FBDG						
Provide a strategy or plan for the implementation of FBDGs						
Allocation of budget for the implementation/dissemination of the FBDG						
Multi-sector involvement (such as health, education, agriculture, etc)						
Adaptability to nutrition and food trends						
Be innovative by developing, applying and adapting new strategies and ideas						
The ability to balance interests and objectives						

On a scale of 1 to 5, how trustworthy do you consider the governmental institution that is responsible for the national dietary guideline in the country you are located in?

Note that 1 is not trustworthy and 5 is very trustworthy.

You can find an overview of national dietary guidelines and responsible governmental institutions from over 100 countries here:
https://www.fao.org/nutrition/education/food-based-dietary-guidelines

1	2	3	4	5	6
Very trustworthy	Trustworthy	Undecided	Slightly trustworthy	Not trustworthy	The country I am located in has no dietary guideline

If 1 or 2 apply:

Which of the following reasons explain your trust in the responsible authority?

- o The scientific committee consists of qualified experts
- o Politicians follow the recommendations of the scientific committee
- o The employees in the responsible authority show that the transformation towards a healthy, sustainable diet is important to them
- o Decisions are made based on the latest scientific evidence
- o They are transparent with the revision process
- o I don't want to answer this question

If 3 applies:

Which of the following reasons explain your undecidedness?

- o I know too little about the process to answer this question
- o I trust them in some aspects, but not in all
- o I don't want to answer this question

If 4 or 5 apply:

Which of the following reasons explain your weakened trust in the responsible authority?

- o The final decision is made by politicians who are primarily interested in their re-election
- o The power of the food industry to influence nutritional guidance is high
- o Cases of corruption within the government
- o The FBDG revision process is not transparent

- o The latest scientific evidence is not taken into account
- o Politicians are not following the recommendations of the scientific committee
- o I don't want to answer this question

Most FBDGs worldwide are outdated and need to be revised. This should be done in fixed regular cycles, for example, every 5 years.

1	2	3	4	5
Strongly disagree	Disagree	Undecided	Agree	Strongly agree

The authority responsible for the FBDGs should be more active on social media to provide science-based information and counteract misinformation.

1	2	3	4	5
Strongly disagree	Disagree	Undecided	Agree	Strongly agree

What do you think are the policy and community factorsthat may affect the adherence to FBDGs? Please select up to 3 factors that are most important in your opinion.

- o Leaders (politicians, government officials, and managers) as role models influencing individuals' beliefs and behaviors.
- o Fiscal policy interventions (e.g. reduced taxes on healthy, sustainable foods such as vegetables and legumes)
- o Nudge interventions in public canteens to promote healthy and sustainable food choices
- o A rule that national FBDGs are mandatory for meals in schools, hospitals, universities, prisons, nurseries, and other public institutions
- o Distance norms between schools and fast food restaurants
- o Change to the agricultural subsidies (to stimulate the production of healthy, sustainable foods)
- o Community-based nutrition programs (focus on households and neighborhoods instead of individuals)
- o More plant-based menu options in restaurants and more plant-based ranges in supermarkets

- Advertising laws and regulations on less healthy and sustainable foods
- Subsidies for food suppliers who adhere to FBDGs
- Front-of-pack food labeling (e.g. Nutriscore or label for FBDG-aligned foods)
- Cooking and nutrition education as a mandatory school subject
- Agricultural and food policies (e.g. Farm Bill)

What should the government do in the future to improve the content of dietary guidelines as well as the adherence to dietary guidelines?
Please select up to 3 aspects that are most important in your opinion.

- Asking the right questions to the scientific committee to work on
- Provide education programs and training to dieticians, nutritionists, and health care professionals.
- Ensure that nutrition is taught more comprehensively in medical schools
- Mandatory continuing professional development for health and nutrition professionals
- Media campaign about dietary guidelines (TV, newspaper, social media)
- Make FBDGs mandatory for public caterers (e.g. in schools, kindergartens, hospitals etc.) to be in line with the national FBDG.
- Launch School feeding programmes based on FBDGs
- Lower prices for healthy, sustainable foods (through taxes or subsidies)
- Hire more people to work in the responsible institution (e.g. Health Ministry or Nutrition Society)
- More funding for the development and revision as well as for the implementation of FBDGs
- Using FBDGs for guiding food system policies
- Make food better available that is recommended in the FBDG (for example through agricultural reforms)
- Revise and update FBDGs on a regular basis
- Countries should review their guidelines from each other (external review process)
- Collaboration with the FAO (Food and Agriculture Organization)
